# A logic-based method to build signaling networks and propose experimental plans

**DOI:** 10.1038/s41598-018-26006-2

**Published:** 2018-05-18

**Authors:** Adrien Rougny, Pauline Gloaguen, Nathalie Langonné, Eric Reiter, Pascale Crépieux, Anne Poupon, Christine Froidevaux

**Affiliations:** 1 Biotechnology Research Institute for Drug Discovery, National Institute of Advanced Industrial Science and Technology (AIST), Aomi, Tokyo, 135-0064 Japan; 2 PRC, INRA, CNRS, Université François Rabelais-Tours, 37380 Nouzilly, France; 3 CNRS; Université François-Rabelais de Tours, UMR 7292, 37032 Tours, France; 4 Laboratoire de Recherche en Informatique UMR CNRS 8623, Université Paris-Sud, Université Paris-Saclay, Orsay Cedex, 91405 France

## Abstract

With the dramatic increase of the diversity and the sheer quantity of biological data generated, the construction of comprehensive signaling networks that include precise mechanisms cannot be carried out manually anymore. In this context, we propose a logic-based method that allows building large signaling networks automatically. Our method is based on a set of expert rules that make explicit the reasoning made by biologists when interpreting experimental results coming from a wide variety of experiment types. These rules allow formulating all the conclusions that can be inferred from a set of experimental results, and thus building all the possible networks that explain these results. Moreover, given an hypothesis, our system proposes experimental plans to carry out in order to validate or invalidate it. To evaluate the performance of our method, we applied our framework to the reconstruction of the FSHR-induced and the EGFR-induced signaling networks. The FSHR is known to induce the transactivation of the EGFR, but very little is known on the resulting FSH- and EGF-dependent network. We built a single network using data underlying both networks. This leads to a new hypothesis on the activation of MEK by p38MAPK, which we validate experimentally. These preliminary results represent a first step in the demonstration of a cross-talk between these two major MAP kinases pathways.

## Introduction

Within a multicellular organism, the behavior of individual cells is partly controlled by hormones that bind to their cognate receptors. Binding of the ligand triggers conformational changes of the receptor’s spatial structure, resulting in the increase or decrease of its affinity for intracellular partners. In the case of G protein-coupled receptors (GPCRs), binding of the extracellular ligand and resulting conformational changes lead to the binding of G proteins and *β*-arrestins to the intracellular domains of the receptor. In turn, this leads to a number of intracellular cascades, activating or inhibiting intracellular proteins, such as the ERK cascade. These cascades lead, among other things, to the activation/inhibition of transcription factors that regulate gene transcription, and to the activation/inhibition of proteins of the translation machinery. Deciphering these networks is done through numerous wet-lab experiments, and in particular the observation of protein phosphorylation, which plays a major role. The representation of these networks is a diagram, usually referred to as the influence graph. As our knowledge of the complexity of signaling pathways has increased, the influence graphs representing them have also grown in size and complexity.

During the past decade, the development of Systems Biology has led to comprehensive reconstructions of large signaling pathways, by gathering results found in the literature^1–4^. These reconstructed pathways have two particularities as compared to diagrams found earlier in the literature. First, rather than simply depicting influences of molecules on each other (e.g. A activates B), these pathways are detailed representations of the molecular mechanisms that come into play (e.g. A phosphorylates B, and phosphorylated B is active). Second, these pathways are now represented using standards, such as the Systems Biology Graphical Notation (SBGN) Process Description language^5^ (PD), which helps their understanding^6^.

The construction of such comprehensive networks has become difficult due to the ever-growing amount of biological data and experimental results. Moreover, these networks are usually not updated with new findings, due to the cost of these updates. Consequently, automated methods to build such networks are highly needed.

Some methods that automatically build molecular networks have already been proposed. A first class of methods relies on literature mining^7–9^. These methods use Natural Language Processing techniques to extract relevant relationships between molecules (such as “A influences the phosphorylation of B”) from scientific papers, either abstracts^7^ or main text^8,9^. These extracted relationships are used to build a molecular network. These methods allow building comprehensive networks taking into account the vast amount of available literature; however, they do not permit to infer new unknown relationships.

Other methods build networks directly from experimental data^10–12^. A number of these methods use statistical inference (e.g. co-expression analysis, Bayesian analysis) to build gene networks from gene expression data^13–16^ or phosphoproteomic data^17–19^. Such techniques are powerful to unveil new relationships hidden deep in the data. However, they function as black boxes, and do not allow to identify the precise data used to build a given relation. Finally, a third category, relying on the use of experimental data, adopts a radically different point of view, by using reasoning techniques to interpret qualitative data^20,21^. Those methods define expert rules that allow interpreting experimental data in order to build gene regulatory networks^20^ or executable models of signaling pathways^21^. These models are reaction networks, as they model different molecular processes (e.g. translocations, state transition processes). However, again, the precise biological knowledge underlying a given inferred relation remains implicit^22^. Hence, such an inference method^21^ does not aim at building molecular networks that could then be interpreted using different semantics (e.g. Ordinary Differential Equations (ODE)), but rather at building models that already carry a modeling semantics. Another current limitation of this method is the lack of rules to infer relationships from perturbation experiments (such as those involving inhibitors, siRNA, knock-outs or mutants), which are widely used in signaling biology.

Herein, we introduce a new logic-based method to build networks of biochemical reactions from raw experimental results. We have built a large set of interpretative logic rules that allow mimicking the reasoning biologists apply when interpreting experimental results. Our expert rules are formalized within first-order logic, which allows to capture the deductive nature of the expert’s reasoning using logical implication. Moreover, this formalism is expressive enough to formalize this reasoning as far as it allows general rules to be instantiated in several ways. These rules allow inferring precise biochemical mechanisms from a wide variety of experiments, including perturbation experiments. They also allow formulating new hypothetical relations that can then be tested experimentally. Using explicit rules, traces of the reasoning used for the inference can be obtained. Meaning that for any relation within the inferred network, the set of experimental data and rules used to build this relation can be consulted by the expert. Those are of particular importance when two potentially contradictory pieces of knowledge are inferred, as these originate from contradictory experimental results. In that case, the user is able to go back to these experimental results, and either resolve the contradiction, or choose to ignore some. Finally, rules were built such that the inference process can be inverted, unveiling an unprecedented feature: the automatic proposition of experimental plans to test given biological hypotheses. We illustrate our method by building the signaling networks triggered by the folliculo-stimulating hormone receptor (FSHR) and the epidermal growth factor receptor (EGFR). Given that the binding of the follicle stimulating hormone (FSH) to its receptor is known to transactivate the EGFR, we also deduce an integrated network from experiments conducted in the study of both individual networks. This led us to the hypothesis that, as a result of FSH stimulation, p38 mitogen-activated protein kinase (p38MAPK) activates dual specificity mitogen-activated protein kinase kinase (MEK), through the transactivation of EGFR. We give first experimental evidences that validate this hypothesis.

## Results and Discussion

### Explicit interpretative rules

Systems Biology mostly focuses on mechanisms: reactions (e.g. post-translational modifications, complexations) and modulations (e.g. catalyzes, inhibitions) of these reactions. This knowledge is usually represented under the form of graphs, where nodes represent the involved molecules, and arcs represent reactions and modulations.

Biologists use implicit deductive reasoning to infer new biological knowledge from experimental results, thus interpreting them. The implicit deductive rules with which they reason follow the generic framework:

R1 $$\begin{array}{c}\,{\rm{IF}}\,{\rm{an}}\,experimental\,result\,{\rm{is}}\,{\rm{observed}}\\ \,\,{\rm{and}}\,{\rm{some}}\,pieces\,of\,biological\,knowledge\,{\rm{hold}}\\ {\rm{THEN}}\,{\rm{new}}\,pieces\,of\,biological\,knowledge\,{\rm{are}}\,{\rm{established}}\,{\rm{or}}\,{\rm{hypothesized}}\end{array}$$

Those rules are general. Of course, a specific experimental result will lead to a specific interpretation, i.e. to an instantiated piece of biological knowledge, but the manner wherewith it is interpreted does not depend on this particular result. Hence rules in the form of (R1) do not apply only to a particular realization of an experiment but rather to some type of experiment, and it is possible to make explicit the general rules used to interpret results obtained from various types of experiments in order to automate the construction of signaling networks.

#### Building the rules

For a given experiment type, we have built one or more interpretative rules by deciphering the molecular processes that come into play while undertaking this experiment. Let us consider a simple phosphorylation assay. In that type of experiment, the quantity of a given target phosphorylated molecule (e.g. phosphorylated extracellular signal-regulated kinase (phospho-ERK)) is compared between a cell lysate obtained from untreated cells (the control), and a cell lysate obtained from cells previously treated with a signal (e.g. an hormone such as FSH). The measurement of the quantity of the target molecule may be carried out by means of an antibody specific to the given phosphorylation or incorporation of radioactive phosphate for example. The only molecular process that comes into play here is the production of the target molecule from another form of that molecule (e.g. phospho-ERK is produced by the phosphorylation of ERK), which is known to the experimenter. Hence, if ERK is more phosphorylated in the presence of FSH as compared to the control, we can conclude that FSH stimulates the phosphorylation of ERK. Of course, it doesn’t mean that this modulation is direct. Conversely, if the phosphorylation of ERK was weaker, we would conclude that FSH inhibits the phosphorylation. Finally, if the phosphorylation levels were identical in both conditions, we would conclude that FSH has no effect on the reaction.

An experimental result obtained from such an experiment can be interpreted by means of the following rule, considering that the detector is an antibody, and that a greater quantity of the target molecule was observed in the treated cells than in the control:

IF a phosphorylation assay in which the signal is a molecule *X*, the target molecule *Y*^*a*^ being detected by an antibody *A* and obtained by transformation of a molecule *Y*, demonstrates a greater quantity of *Y*^*a*^ in cells treated by *X* than in the control cells, THEN *X* stimulates the process that transforms *Y* into *Y*^*a*^.

This interpretative rule can be formalized in first-order logic as follows:

R2 $$\begin{array}{l}\,{\rm{IF}}\,pa(X,Y,A,increase)\\ \,\,{\rm{and}}\,antibodyAgainst(A,{Y}^{a})\,and\,{mod}ifiedForm({Y}^{a},Y)\\ {\rm{THEN}}\,{modulates}(X,Y,{Y}^{a},increase,unknown,confirmed)\end{array}$$

The left-hand side of the rule (before the “THEN”) is called its *premises*, while its right-hand side is called its *conclusion*. The experimental result is expressed in the premises, while its interpretation is given in the conclusion. Such deductive rules should be read as follows: IF the premises hold, THEN the conclusion holds.

Lowercase symbols before the opening parenthesis (e.g. *pa* (phosphorylation assay) or *modulates*) are predicate symbols that express relations between molecules or parameters. Their arguments (here, variables and constants) are contained between the associated parentheses: lowercase symbols (e.g. *increase*, *confirmed*) are *constants* that refer to molecules or specific parameters, while uppercase symbols (e.g. *X*, *A*) are *variables* that refer to any molecule or parameter value, and can be instantiated by constants. A fully instantiated predicate (i.e. with all its variables replaced by constants), such as *modulates*(*fsh*, *erk*, *perk*, *increase*, *unknown*, *confirmed*), is called a *fact*.

The predicate symbol *modulates* takes six arguments, and *modulates* (*X*, *Y*, *Y*^*a*^, *E*, *D*, *S*) qualifies the modulation by molecule *X* of the process that transforms molecule *Y* into molecule *Y*^*a*^. The three additional arguments are parameters of this modulation:

- *E* is the *effect* parameter. It stands for the effect of the modulation. It can take three values: *increase*, *decrease*, or *noeffect*, depending on whether the modulation is a stimulation, an inhibition or absent, respectively. This parameter is also an argument of predicates that express experimental results such as *pa*. For those, *increase*, *decrease* and *noeffect* constants indicate a higher, a lower and an equal quantity of a target molecule in the treated cells than in the control cells, respectively.
- *D* is the *distance* parameter. It stands for the distance with which the modulation takes place: a direct modulation (i.e. with no intermediaries) is represented by the *direct* constant, and an indirect stimulation by the *indirect* constant. As for the *unknown* constant, it indicates that the distance with which the modulation takes place is unknown.
- *S* is the *status* parameter. It can take one of the three values *confirmed*, *hypothesis* and *infirmed*. The *confirmed* constant indicates that the modulation has been experimentally validated; the *hypothesis* constant indicates that the modulation is hypothetical, and should be proved by further experiments; and the *infirmed* constant indicates that the modulation has been experimentally invalidated.

#### Different interpretative rules for different types of experiments

Although the number of different experiment types that exist is large, the vast majority of experiments conducted in Systems Biology studies belong to a reduced list. We have gathered 28 different experiment types that were used to establish a comprehensive map of the FSHR-induced network^4^. Table 1 shows the different types of experiments taken into account. A complete description of the various types of experiments is given in *Listing of predicates* in Supplementary Note.

**Table 1:** All types of experiments that can be interpreted by the set of expert rules.

Type of the conclusion	Method	Category	Disruptor	Detection method	Experiment type	Experimental result predicate	Conceptual experiment type predicate
Modulation	Dosage	Enzymatic assay		Radio	25EA	*ea*(*X*, *S*1, *S*2, *E*)	*simpleEa*(*X*, *S*1, *S*2, *E*)
			Antagonist		50ACPEA	*acpea*(*X*, *S*1, *S*2, *I*, *E*)	*complexEa*(*X*, *S*1, *S*2, *I*, *E*)
			Inhibitor		50ICPEA	*icpea*(*X*, *S*1, *S*2, *I*, *E*)	
			siRNA		75SCPEA	*scpea*(*X*, *S*1, *S*2, *I*, *E*)	
		Phosphorylation assay		Antibody	25PA	*pa*(*X*, *Y*, *A*, *E*)	*simplePa*(*X*, *Y*, *A*, *E*)
				Radio	25PRA	*pra*(*X*, *Y*, *A*, *E*)	
			Antagonist	Antibody	50ACPPA	*acppa*(*X*, *Y*, *A*, *I*, *E*)	*complexPa*(*X*, *Y*, *A*, *I*, *E*)
			Inhibitor		50ICPPA	*icppa*(*X*, *Y*, *A*, *I*, *E*)	
				Radio	50ICPPRA	*icppra*(*X*, *Y*, *A*, *I*, *E*)	
			siRNA	Antibody	75SCPPA	*scppa*(*X*, *Y*, *A*, *I*, *E*)	
		ELISA		Antibody	25ELISA	*elisa*(*X*, *A*, *E*)	*simpleElisa*(*X*, *A*, *E*)
			Inhibitor		50ICELISA	*icelisa*(*X*, *A*, *I*, *E*)	*complexElisa*(*X*, *A*, *I*, *E*)
			siRNA		75SCELISA	*scelisa*(*X*, *A*, *I*, *E*)	
		Radio-immunology Experiment		Radio	25RIA	*ria*(*X*, *A*, *E*)	*simpleRia*(*X*, *A*, *E*)
			Inhibitor		50ICRIA	*icria*(*X*, *A*, *I*, *E*)	*complexRia*(*X*, *A*, *I*, *E*)
		Western Blot		Antibody	25WB	*wb*(*X*, *A*, *E*)	*simpleWb*(*X*, *A*, *E*)
			Inhibitor		50ICWB	*icWb*(*X*, *A*, *E*)	*complexWb*(*X*, *A*, *I*, *E*)
					75RIWB	*riWb*(*X*, *A*, *E*)	
		QRTPCR			25RTPCR	*qrtpcr*(*X*, *Y*, *E*)	*simpleQrtpcr*(*X*, *Y*, *E*)
			Inhibitor		50ICQRTPCR	*icqrtpcr*(*X*, *Y*, *I*, *E*)	*complexQrtpcr*(*X*, *Y*, *I*, *E*)
Interaction	Precipitation	IP			25IP	*ip*(*X*, *S*1)	*oneToManyInteractionExp*(*X*, *S*1)
	Pulldown	GSTPulldown			25GSTPULLDOWN	*gstPulldown*(*X*, *S*1)	
		BDPulldown			25BDPULLDOWN	*bdPulldown*(*X*, *S*1)	
	FRET	FRET			50FRET	*fret*(*X*, *Y*)	*oneToOneInteractionExp*(*X*, *Y*)
	Cristallography	Cristallography			1003D	*threeD*(*S*1)	*manyInteractionExp*(*S*1)
	Precipitation	IP			125IPD	*ipd*(*X*, *Y*, *S*1, *E*)	*effectOnOneToManyInteractionExp*(*X*, *Y*, *S*1, *E*)
Localization	Fluorescence Coloration				25FLUO 25IHC	*fluo*(*A*, *C*, *E*)*ihc*(*A*, *C*, *E*)	*detectionExp*(*A*, *C*, *E*)

Experiments that can be interpreted by the same general rule have the same color.

All these experiments can be classified into three main groups depending on the kind of biological knowledge they allow to deduce (see first column of Table 1). All experiment types can be further classified according to different additional criteria, such as the experimental method they bring to play, or the eventual disruptor they use (see Table 1 for more details). This last criterion allows to distinguish between simple assays (e.g. a phosphorylation assay (*pa*)) and their corresponding complex assays that are realized in presence of a disruptor (e.g. an inhibitor in the case of an *icppa*). Based on this classification, we generalized the rules related to a number of experiment types taken into account (see *Rule generalization* in Supplementary Note for some example). In Table 1, all the experiment types that can be interpreted by the same general rules have the same color. All the rules are given in *Listing of rules* in Supplementary Note.

#### Simple and complex interpretative rules

In (R2), premises are formed of predicates that cannot be deduced by any other interpretative rule: we call them *background knowledge* predicates. As for the conclusion, it is formed of a single predicate that provides deduced pieces of knowledge: we call it *deduced* predicate. We distinguish two kinds of rules, depending on whether they only use background knowledge in their premises, and thus do not require any information deduced from another rule (*simple rules*), or on the contrary cannot be interpreted in the absence of anterior knowledge (*complex rules*). Rule (R2) is an example of simple rule. All the rules built to interpret experiments that involve inhibitors or antagonists are examples of complex rules, as they cannot be interpreted without the results of the same experiment conducted without the inhibitor or antagonist.

Let us consider the experiment that consists in comparing the phosphorylation of ERK induced by the FSH between untreated cells and cells treated with an inhibitor of protein kinase A (PKA) (for example, with the H89 inhibitor^23^). The result of the experiment is that the phosphorylation of ERK induced by FSH is hampered by the inhibitor, from which an expert can make the hypothesis that PKA might be an intermediary of the activation of ERK by FSH. This type of experiment is called an *icppa*, and can be interpreted by the following rule when a *decrease* effect is observed:

R3 $$\begin{array}{c}\,\,{\rm{IF}}\,{iccpa}({X}^{a},Y,A,I,{decrease})\\ \,\,{\rm{and}}\,{antibodyAgainst}(A,{Z}^{a})\,{\rm{and}}\,{inhibitorAgainst}(I,{Y}^{a})\\ \,\,{\rm{and}}\,{modifiedForm}({Y}^{a},Y)\\ \,\,{\rm{and}}\,{notModified}({{X}}^{a})\\ \,\,{\rm{and}}\,{modulates}({X}^{a},Z,{Z}^{a},{increase},{unknown},{confirmed})\\ {\rm{THEN}}\,{modulates}({Y}^{a},Z,{Z}^{a},{increase},{unknown},{confirmed})\\ \,\,{\rm{and}}\,{modulates}({X}^{a},Y,{Y}^{a},{increase},{unknown},{hypothesis})\end{array}$$

In this type of experiment, the signal is the molecule *X*^*a*^ (FSH in our example), and the corresponding rule is triggered only if *X*^*a*^ has no modified form, as the predicate *notModified* (*X*^*a*^) appears in its premises. Biologically, this means that *X*^*a*^ is not modified in the reaction, which is the case for an extracellular hormone such as FSH, for example. From this type of experiment, we conclude that *Y*^*a*^ (activated PKA) stimulates the transformation of *Z* (ERK) into *Z*^*a*^ (phospho-ERK), since the quantity of *Z*^*a*^ is decreased in presence of the inhibitor of *Y*^*a*^ (H89). From this first conclusion, two alternatives appear: either *Y*^*a*^ can influence *Z*^*a*^ independently from *X*^*a*^ (activated PKA stimulates the phosphorylation of ERK independently of FSH) and no other additional conclusion has to be made, or *Y*^*a*^ is an intermediary of the induction of *Z*^*a*^ by *X*^*a*^, meaning that additionally, *X*^*a*^ stimulates the transformation of *Y* into *Y*^*a*^ (FSH stimulates the activation of PKA). As we cannot choose between the two alternatives with only that experiment, both are considered as hypothetical until further confirmation, hence the *hypothesis* status for the additional conclusion of the second alternative.

The following rule allows interpreting the same type of experiment, but leaving the possibility that *X*^*a*^ is the modified form of its inactive counterpart *X*:

R4 $$\begin{array}{c}\,\,{\rm{IF}}\,{icppa}({X}^{a},Y,A,I,\mathrm{decrease})\\ \,\,{\rm{and}}\,{antibodyAgainst}(A,{Z}^{a})\,{\rm{and}}\,{inhibitorAgainst}(I,{Y}^{a})\\ \,\,{\rm{and}}\,{modifiedForm}({Y}^{a},Y)\\ \,\,{\rm{and}}\,{modifiedForm}({X}^{a},X)\\ \,\,{\rm{and}}\,{modulates}({X}^{a},Z,{Z}^{a},{increase},{unknown},{confirmed})\\ {\rm{THEN}}\,{modulates}({Y}^{a},Z,{Z}^{a},{increase},{unknown},{confirmed})\\ \,\,{\rm{and}}\,{modulates}({X}^{a},Y,{Y}^{a},{increase},{unknown},{hypothesis})\\ \,\,{\rm{and}}\,{modulates}({Y}^{a},X,{X}^{a},{increase},{unknown},{hypothesis})\end{array}$$

This rule has one more conclusion compared to rule (R3):

$${modulates}({Y}^{a},X,{X}^{a},{increase},{unknown},{hypothesis})$$

The two alternatives given for the case where *X*^*a*^ has no modified form remain valid, but there is a third one: *X*^*a*^ might be an intermediary of the induction of *Z*^*a*^ by *Y*^*a*^, and thus *Y*^*a*^ might stimulate the process that transforms *X* into *X*^*a*^. As for the previous case, we cannot rule out any alternative.

**Figure 1:**
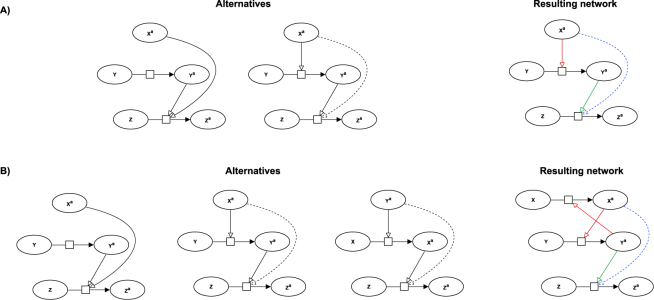
Interpretation of a phosphorylation assay with an inhibitor. (**A**) Case where *X*^*a*^ has no modified form, treated by rule (R3). In the first alternative (left), *Y*^*a*^ influences the transformation independently from *X*^*a*^; in the second alternative (right), *Y*^*a*^ is an intermediary of the induction of *Z*^*a*^ by *X*^*a*^. (**B**) Case where *X*^*a*^ has a modified form *X*, treated by rule (R4). In the first alternative (left), *Y*^*a*^ influences the transformation independently from *X*^*a*^; in the second alternative (middle), *Y*^*a*^ is an intermediary of the induction of *Z*^*a*^ by *X*^*a*^; in the last alternative (right), *X*^*a*^ is an intermediary of the induction of *Z*^*a*^ by *Y*^*a*^. For both cases, the confirmed or hypothetical facts that result from the superposition of the different alternatives are represented under the form of a resulting network, on the right of the figure. Blue modulations represent premises, green modulations conclusions that are confirmed, red ones hypotheses, and dashed ones conclusions that could be indirect and explained by transitivity.

Figure 1 shows the interpretation made from an *icppa* in the case where the signal has as no modified form (top) and in the case where it has one (bottom). For the case where *X*^*a*^ has a modified form, the network on the left represents the alternative where *Y*^*a*^ is an intermediary of the induction of *Z*^*a*^ by *X*^*a*^, and the network on the right the alternative where it is *X*^*a*^ that is an intermediary between *Y*^*a*^ and *Z*^*a*^.

### Analytical rules

In addition to interpretative rules, we have built a set of analytical rules that do not interpret any experimental result, but allow to either complete the background knowledge, or to deduce new pieces of knowledge or hypotheses. For example, rule (R5) allows generating an hypothetical modulation based on the distance of already known modulations. Its meaning is the following: if *X*^*a*^ modulates a process that transforms *Z* into *Z*^*a*^, *Y*^*a*^ modulates the same process but with a direct distance (i.e. with no intermediary coming into play), and *Y*^*a*^ is the modified form of *Y*, then *X*^*a*^ might modulate the process that transforms *Y* into *Y*^*a*^.

R5 $$\begin{array}{c}\,\,{\rm{IF}}\,{modulates}({X}^{a},Z,{Z}^{a},{increase},{unknown},{confirmed})\\ \,\,{\rm{and}}\,{modulates}({Y}^{a},Z,{Z}^{a},{increase},{direct},{confirmed})\\ \,\,{\rm{and}}\,{modifiedForm}({Y}^{a},Y)\\ {\rm{THEN}}\,{modulates}({X}^{a},Y,{Y}^{a},{increase},{unknown},{hypothesis})\end{array}$$

Here, we make the hypothesis that *Y* is an intermediary of the modulation of *Z* by *X*. We do not hypothesize that it is *X* that is an intermediary between *Y* and *Z*, since we know that *Y* has a direct effect on *Z*.

### Two tasks: building networks and inferring experimental plans

#### Automated construction of cell signaling networks via deduction

The set of interpretative and analytical rules we propose allows establishing automatically new pieces of knowledge from a number of given experimental results. More precisely, given a set of background facts and a set of experimental facts, these rules allow *deducing* new facts that can then be represented under the form of a signaling network (using the SBGN PD language^5^ for example). A step by step example of such a deductive task is given in *Example of deduction* in Supplementary Note.

By using formal explicit rules and facts, one can track the different deduction steps that lead to a particular fact, in a way that is understandable, and thus verifiable, by the biologist. For a deduced relation, the set of experimental results and rules that enabled its deduction is called its *trace*. Traces are especially useful when automated reasoning leads to facts that could seem contradictory. For example, two facts, one qualifying the stimulation of a process by a molecule and the other the inhibition of that same process by the same molecule can sometimes be obtained. This is quite possible, for example if two different pathways exist, leading to the same reaction, one being inhibitory and the other activatory. However, experimental results that lead to such a pair of facts should be carefully verified, since they could also result from erroneous experiments. In this case, thanks to traces, our system allows the user going back to these two results, and possibly choosing to ignore one.

#### Automated proposition of experimental plans via abduction

Our method also allows to go further. It is often the case that a given molecule or reaction is expected to participate in a given signaling network, and the question then arises of choosing experiments that could validate this hypothesis. Our approach provides a way of answering this question through a method of reasoning called *abduction*, that can be seen as the inverse process of deduction, and whose goal is to find minimal explanations of an observation, given a background logical theory.

In order to check a given biological hypothesis, we first assume that this hypothesis is true, and formalize this assumption by a fact with the status *confirmed*. Then, using abductive reasoning, we obtain minimal alternative sets of experimental results, each set alone being sufficient to explain the biological fact. The obtained set of inferred experimental results constitutes an experimental plan, that can be carried out in order to test the input hypothesis. A step by step example of such an abductive task is given in *Example of abduction* in Supplementary Note.

### Application to the FSHR-induced and the EGFR-induced signaling networks

We applied our method to the network induced by the FSHR, and to the network induced by the EGFR. FSH is a pituitary hormone of major importance in the control of male and female reproduction. It acts *via* its cognate receptor, the FSHR, a Gs-coupled seven transmembrane domain receptor. In females, FSH participates, *inter alia*, in the induction of ovarian follicles’ growth and maturation. In males, it participates in the induction of spermatogenesis by stimulating Sertoli cells. When FSH binds to its receptor, the latter triggers several signaling pathways: the G-dependent pathway, leading to the production of second messenger cyclic AMP (cAMP), but also the phosphatidylinositol-4,5-bisphosphate 3-kinase (PI3K)-dependent and *β*-arrestin-dependent pathways. As for the epidermal growth factor (EGF), it participates in the tissue growth. It acts via its cognate receptor (the EGFR), which belongs to the tyrosine kinase receptors (RTK) family. Activation of EGFR triggers three main signaling pathways: the ERK pathway, the PI3K/protein kinase B (Akt) pathway, and the Jakus kinase (JAK)/signal transducer and activator of transcription protein (STAT) pathway. Finally, these two networks are not independent of each other: the EGFR is transactivated by the FSHR induced network, via the Rous sarcoma oncogene (Src) (see^4^ for a review).

We first built the FSHR induced network, by automatically interpreting a set of experimental results linked to this network, which had been used in a previous review^4^. We then automatically interpreted this set of experimental results by augmenting it with another set of results related to the EGFR induced network. This last interpretation allowed discovering a new hypothesis, for which we subsequently acquired experimental evidence.

#### Retrieving experimental and background knowledge facts

The set of experimental and background knowledge facts related to both networks was retrieved from the literature (see Methods for further details). For the FSHR induced network, 191 experimental results were retrieved, whereas this number reached 274 for the EGFR induced network. In total, 1448 background facts were also retrieved for both networks together.

#### Automatic inference of the FSHR induced network

We performed the interpretation of the 191 experimental results related to the FSHR induced network, using the set of expert rules and the set of background knowledge facts. We obtained 1535 new facts. Among them, 1184 facts corresponded to background knowledge facts or modulations with a *noeffect* parameter status. The remaining 351 pertinent facts (i.e. formalizing a process, the modulation of a process or the localization of a molecule) formed together a molecular network. We compared this network to the FSHR induced network that we previously built manually using the same set of experiments^4^ (see Supplementary Fig. S1 for an overview of this network), which will be considered as the reference network. We give an overview of the result of this comparison for the G protein pathway in *Comparison for the G protein pathway* Supplementary Note.

Most differences observed between the two networks can be explained, either by the fact that in graphical representations some details are omitted for the sake of clarity, or by the presence of knowledge in the reference network transferred from other networks, mostly from signaling networks triggered by other GPCRs. This tends to show that our expert rules are complete enough to deduce all the main processes and modulations of a given network.

We conducted a similar work on the signaling network of the EGFR (Epidermal Growth Factor Receptor). In that case, our reference was the network of the Pathway Interaction Database (PID)^24^. Similar to the FSHR case, we extracted the experimental facts from the literature cited in the PID network, and ran the inference. Comparison of the inferred and reference networks showed no significant differences.

#### Discovering a new hypothesis on the phosphorylation of MEK by p38MAPK

Because of its complexity, a signaling network is often not studied as a whole, but through the study of individual pathways. However, these different pathways are not independent of each other, and the cellular outcome of the stimulation results from the precise timing and intensity of their activation. Consequently, new knowledge would certainly arise from the study of the complete network, which is possible only through automated modeling. To illustrate this, and because EGFR is known to be transactivated by FSHR, we decided to merge the sets of experimental and background knowledge facts related to the FSHR and the EGFR. We then used our expert rules to deduce new facts from this set. As expected, none of the new deduced facts was obtained by using interpretative rules: indeed, all experimental facts had already been used to deduce facts for each of the two networks. However, rule (R5) allowed generating new hypotheses that were not generated by either of the two sets of experimental results alone. Eleven such new facts were found. Although many of them are not really useful for understanding the biological outcome of the stimulation, one interesting hypothesis emerging from this analysis is that phosphorylated p38MAPK could activate MEK:

F1 $$modulates(pp38mapk,mek,pmek,increase,unknown,hypothesis)$$

This hypothesis is deeply interesting for two main reasons. First, p38MAPK is known as the target of stress-activated MAP2K (MAP2K3, MAP2K4 and MAP2K6 in particular), whereas MEK targets MAPK3 (among which ERK) involved in growth. Establishing the existence of a cross-talk between the stress and growth MAPK pathways would show that the two systems are not as insulated as generally thought. Moreover, in signaling cascades, a MAP2K usually activates a MAPK. But here, the hypothesis is that a MAPK (p38) activates a MAP2K (MEK). Second, validating this hypothesis would allow to validate at the same time a number of other hypotheses also generated using rule (R5), that relate to modulations of the phosphorylation of MEK by molecules upstream of phospho-p38MAPK. This gives more weight to such hypotheses, and illustrates one advantage of automatic reasoning: after a small change in the whole theory (here, the addition of a new modulation), new pieces of knowledge that are a consequence of this change can easily be deduced, even if the new pieces of knowledge are located far from where the change is initially made within the network.

#### Experimental plans to establish the phosphorylation of MEK by phospho-p38MAPK in the FSHR system

Having deduced the hypothesis that MEK may be phosphorylated by phospho-p38MAPK (fact (F1)), we inferred automatically all experimental plans that would allow validating it. We obtained six different experimental plans, each containing only one experiment to carry out. Those six plans were derived from the following abduced facts:

- *icpea*(*pmek*, *s*_*1*_*erk1*, *s*_*1*_*perk1*, *sb203580*, *decrease*)
- *icpea*(*pmek*, *s*_*1*_*erk2*, *s*_*1*_*perk2*, s*b203580*, *decrease*)
- *icria*(*fsh*, *r*_*pmek*, *sb203580*, *decrease*)
- *icelisa*(*fsh*, *a*_*pmek*, *sb203580*, *decrease*)
- *pa*(*pp38mapk*, *mek*, *a*_*pmek*, *increase*)
- *icppa*(*fsh*, *mek*, *a*_*pmek*, *sb203580*, *decrease*)

Experimental plans containing the *icria*, the *icelisa* and the *icppa* predicates proposed to study the effect of an inhibitor of p38MAPK (here, SB203580, which has been shown to be extremely specific, and has no effect on MEK and ERK activities^25^) on the stimulation of FSH-triggered MEK phosphorylation using a radio-immunologic assay, a phosphorylation assay or an ELISA, respectively. The two *icpea* plans proposed to study the effect of the exact same inhibitor on the activity of MEK, through the study *in vitro* of ERK phosphorylation. Finally, the plan with the *pa* proposed to test directly the effect of phosphorylated p38MAPK on MEK phosphorylation using a phosphorylation assay.

**Figure 2:**
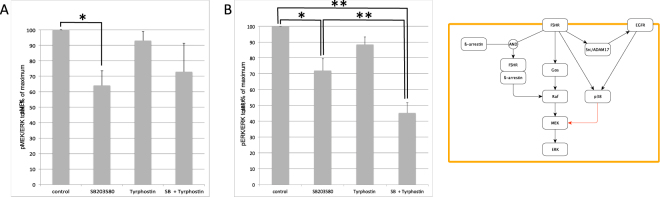
(**A**) Levels of MEK and (**B**) levels of ERK phosphorylation in HEK293 cells expressing the FSHR 10 min after activation by FSH in the presence or absence of SB203580 (inhibitor of p38MAPK) and presence or absence of tyrphostin (inhibitor of EGFR). Significant differences are labeled with stars. Results are expressed as the % of the maximum response elicited by FSH in the absence of any inhibitor. (**C**) The SBGN AF^43^ map representing the different pathways triggered by the FSHR, as well as the transactivation of the EGFR via Src. Our confirmed hypothesis, that p38MAPK stimulates the phosphorylation of MEK, is colored in red.

We carried out the simplest plan to implement, which was the *icppa*. This plan proposed to inhibit p38MAPK and to observe the phosphorylation of MEK in stimulated cells using an antibody specific to the phosphorylated (active) form of MEK, and to compare with activated cells in the absence of the inhibitor. The results (Fig. 2) confirm the hypothesis: p38MAPK stimulates the phophorylation of MEK, and through it, the phosphorylation of ERK. Indeed, the phosphorylation level of MEK is significantly lower in the presence of SB203580, an inhibitor of p38MAPK, than in the control conditions. The same phenomenon is observed for the phosphorylation level of ERK.

As this hypothesis did not arise from the study of the FSHR signaling network alone, it was also interesting to test whether this modulation arose through the transactivation of EGFR, or was an unknown consequence of the EGFR-independent FSHR signaling pathway. For this purpose we repeated the experiment in the presence of an inhibitor of the EGFR. Comparing the levels of MEK or ERK phosphorylation in the presence of Tyrphostin, the inhibitor of EGFR, shows a tendency to decrease, but this effect is not significant. Comparing the phosphorylation of ERK is the presence of both Tyrphostin and SB203580 shows a significant decrease as compared to control conditions, but also to the conditions with SB203580 alone. Moreover, this is not observed for MEK phosphorylation levels. This could mean that there exists a pathway leading to ERK phosphorylation which depends on EGFR (since the effect is decreased in the presence of Tyrphostin), but neither on p38MAPK (since SB203580 is present in both conditions), nor on MEK (since the effect is not observed for MEK).

However, the observed differences could also be due to technical reasons, such as differences in quality or affinity of the antibodies used to detect the phosphorylated forms of MEK and ERK, or differences in the total amounts of both proteins present in the cells. Drawing more definitive conclusions would require more experiments, using different protocols, which was not the scope of the present paper.

### From deduced facts to mathematical models

The output of our method is a set of logical deduced facts that formalize knowledge on molecular mechanisms, and that form together a molecular network. These facts can easily be translated to standard graphical or textual languages, such as SBGN PD^5^/SBGN ER^26^ or BioPax^27^. Also, the output networks represent static knowledge and do not carry any dynamics semantics. Hence, they can be used as inputs for (automatic) mathematical model building under a wide variety of semantics and formalisms, such as the rule-based entity-centered semantics and ODEs^9,28,29^, plain ODEs^30^, or qualitative semantics^29,31^. These mathematical models can in turn be stored and exchanged using standard formats such as the BioNetGen Language format^28^ for rule-based models, or the Systems Biology Markup Language format^32^ for ODEs and qualitative models.

### Scalability and automation

One weakness of our framework that clearly appeared during this work is the manual entry of experimental and background facts. Going through scientific papers to extract experimental results and formating them for the inference engine is a tedious and error-prone task. Consequently, scalability of the method, apart from pure computational aspects, will also need the automation of this extraction and formating task. This led us to start the development of a method, using natural language processing, to automate this task. We have already shown that we can isolate, within a scientific paper, the sentences relative to the results of an experiment^33^. We are now developing cascades within the CasSys system^34^ to format these experimental results as facts that could be used directly within the framework described in the present paper. The first tests have shown that we were able to extract and format successfully both background and experimental facts with good precision (76%) and recall (88%).

This work is still preliminary, and will require many more developments. However, we already have a proof of concept that such automated extraction and formatting of background and experimental facts from literature is feasible using natural language processing.

Another question is how scalable is the method? Giving a definitive answer to this question would require a very large dataset of experimental facts, which will be available only after completion of the natural language treatment-based extraction and formatting method. However, the computation times observed during our first tests seem to depend linearly on the number of experimental facts (Table 2). Similarly, the number of deduced facts seems to depend linearly on the number of experimental facts. Finally, the computation times are very low, and if the linearity is confirmed, the framework could be applied to very large datasets.

**Table 2:** Numbers of experimental facts, deduced facts and computation times for the studied networks.

Network	FSHR	EGFR	FSHR + EGFR
Experimental facts	191	274	465
New facts	1552	2413	3058
Pertinent facts	344	583	993
CPU time (s)	4.4	8.7	12.8

### Knowledge transfer

As discussed above, in the signaling network we built, some relations were missing because they have not been demonstrated in the case of the FSHR and/or the EGFR. However, these relations are considered as true by the experts, either because they have been demonstrated using purified proteins, or because they have been demonstrated for many different conditions.

As an example, when we reconstructed the EGFR signaling network, we did not obtain the inhibition relation between Tsc2 and Rheb. Indeed, in the scientific paper corpus we have used, this inhibition of Rheb by Tsc2 was not demonstrated. We then searched the literature for such a demonstration, but we could not find an experiment demonstrating that EGF-activated Tsc2 inhibits Rheb. However, we found many papers demonstrating this inhibition relation, using purified proteins^35^ or *in vivo*, following different stimulations such as insulin^36^, ephrin^37^ or tissue plasminogen activator^38^. More generally, Tsc2 has been shown to be an important hub in the signaling pathways triggered by many different signals such as amino acid availability, growth factors, cytokines, and energy^39^. Thus, in this particular example, it seems reasonable to consider that in the EGFR pathway Rheb is inhibited by Tsc2.

This raises the question of transferring knowledge between different networks and/or different biological conditions. The example above is a direct action, demonstrated using purified proteins. In such cases we could consider that in any cell, and under any condition, if the two proteins are present, the conclusion holds. But many possible cases are more difficult to decide than this one. For example, can experimental data obtained in a given cell line be used to build a network in another cell line? Or when the cell lines are the same, but the experimental conditions, such as growth media, are different? These are of course questions that cannot be answered only through computational tools, and it will ultimately be the user’s decision. However, tools can be designed to guide this decision. In our framework the user can always come back to the experimental data used to deduce a given relation. This can be such a guiding element, but more will have to be added in the future.

## Conclusions

In this article, we propose a logic-based method to automate the construction of signaling networks. Our method tries to reproduce the reasoning made by biologists when interpreting experimental results, by automatically deducing new knowledge using a set of expert rules that make explicit this reasoning. These rules allow interpreting experimental results coming from a large variety of experiments that are usually employed in cell signaling, and to infer precise mechanisms, at the molecular reaction scale. Moreover, they allow formulating a number of biological hypotheses, and experimental plans to validate them. Our method allowed us to successfully reconstruct two large networks: the FSH-triggered and the EGF-triggered networks. Using the two sets of underlying data at the same time led us to formulate the hypothesis that p38MAPK would stimulate the phosphorylation of MEK. In order to validate this hypothesis, we automatically inferred an experimental plan using our method, that we have carried out. The results obtained show that p38MAPK stimulates the phosphorylation of MEK in an FSHR-induced system, as MEK activation is significantly lower in the presence of p38MAPK inhibitor. As a consequence, ERK phosphorylation is also decreased when p38MAPK is inhibited. We also observed further inhibition of activity in the presence of the EGFR inhibitor for ERK, but not for MEK. This could be the effect of another activation mechanism of ERK, depending on EGFR transactivation, but not on p38MAPK or MEK. This first experimental validation would need to be consolidated, which is not the object of the present paper. However, it constitutes a first step in the discovery of a yet-unknown cross-talk between these two very important signaling pathways.

Although preliminary, these results demonstrate that our method allows to make high-level hypotheses, based on already known facts, and could be an important driving force for understanding the signaling pathways in their complexity. Applying our method to large sets of experimental data could be facilitated by new evolutions, and our efforts will take two directions. First, we need to expand the number of experiments that can be automatically interpreted. In particular, experiments involving the use of mutants (either cells expressing mutant proteins, or even knock-out animals) are often used, but the corresponding rules are delicate to establish, since the driving idea for making a mutation is often implicit. Another important area we have to formalize corresponds to the *in vivo* experiments. Second, we have to be able to extract the facts automatically from scientific papers. Indeed, manually gathering the relevant literature, extracting the experimental data, and subsequently writing the facts is a tedious task, and consequently limits the applicability of the method. A standard format for reporting experimental results would of course greatly facilitate automated extraction, and the work discussed here could be straightforwardly used to design such a format. However, we do not believe scientific community is ready to format and report experimental results, and not enough results would be gathered by this mean to make the method really powerful. Instead, we are developing a method, based on natural language processing, which should allow to extract and format experimental facts directly from published papers.

## Methods

### Retrieving the experimental facts

Experimental facts relative to both networks have been manually retrieved from the literature. The corpus of scientific papers considered for FSHR signaling was the exact same one used to build a comprehensive literature network^4^, so that the inferred network and the literature network could be compared. This corpus was initially assembled by reviewing the literature. As for the corpus of scientific papers considered for EGFR signaling, it has been found in the PID^24^ (data now available through the NDEx database^40^).

### Deduction with answer set programming

Deduction was performed by means of answer set programming (ASP) using the clingo software^41^. Our expert rules were straightforwardly translated into ASP rules. The ASP program consists of a set of background knowledge and experimental facts, and the translated expert rules. This program has a unique answer set which is exactly the set of facts that can be deduced from the initial facts and the rules. Traces can be obtained at the same time as sets of deduced facts by using a simple transformation of the ASP rules (see *Traces in ASP* in Supplementary Note for more details). Computations were made on an Intel(R) Xeon(R) CPU E5-1620v4 @ 3.50 GHz.

### Inference of experimental plans with SOLAR

Inference of experimental plans was performed with the software SOLAR^42^, using abductive reasoning. As for deduction with ASP, the expert rules were straightforwardly translated into SOLAR’s input language. Experimental plans for a given hypothesis were then computed using the negation of the hypothesis as the top clause.

### Cell Culture and Western-blots

Human embryonic kidney (HEK)-293 cells stably expressing mFSH-R were cultured in minimum Eagle’s medium supplemented with 10% fetal bovine serum and 1% penicillin/streptomycin in 100-mm dishes. Slow growing early passage (<10) cells were divided into 12-well plates and, at 50% confluency, serum-starved for 4 h prior to stimulation. Cells were then incubated with SB203580 (10 *μ*M, 30 min) (Sigma, St Louis, MO), Tyrphostin (10 *μ*M, 1 h) (Sigma) or the combination of the 2 inhibitors, before stimulation with 100 ng/ml (3.3 nM) of recombinant human FSH (Merck Serono, Darmstadt, Germany). Cell lysates were done in 2 × SDS-sample buffer (pH 6.8), followed by boiling. Equivalent amounts of proteins were separated by SDS/PAGE on Tris-glycine polyacrylamide gels, transferred to nitrocellulose membranes, and immunoblotted with rabbit polyclonal antibodies. The anti- phospho (T202/Y204, diluted 1:2,000) and total (diluted 1:6,000) ERK 1/2 antibodies were from Cell Signaling Technology (Beverly, MA) and Santa Cruz (Dallas, TX), respectively. Rabbit anti-phospho-MEK 1/2 (S217/S221, diluted 1:1,000) and mouse anti-total MEK 1/2 (diluted 1:2,000) antibodies were from Cell Signaling Technology (Beverly, MA). Goat anti-rabbit IRDye® 680 antibody (Licor®, Lincoln, NE) was used for detection with an Odyssey® CLx scanner (Licor®) and signals were quantified using Image Studio software (Licor®). Normalization was applied as ratio of phospho ERK/total ERK signals. Data were analyzed using the GraphPad PRISM software and differences between two groups were assessed by a two-tailed paired t-test. The cut-off for significance is either 0.05 (⋆) or 0.001 (⋆⋆).

### Data and scripts

All data and scripts that we used to establish the results presented in this document are available at: http://bios.tours.inra.fr/bios_group/sites/bios_group/IMG/zip/archive_article.zip.

## Electronic supplementary material

Supplementary notes
