# Supplementary material for: A logic-based method to build signaling networks and propose experimental plans
Source: Sci Rep. 2018 May 18;8:7830. doi: 10.1038/s41598-018-26006-2 (PMC5959848; doi:10.1038/s41598-018-26006-2)
Supplement: Supplementary file 1 — Supplementary notes [file 41598_2018_26006_MOESM1_ESM.pdf]

Supplementary notes and figure to *A logic-based method to build signaling networks and propose experimental plans*

Adrien Rougny[1,4], Pauline Gloaguen[2], Nathalie Langonné[2,3], Éric Reiter[2], Pascale Crépieux[2], Anne Poupon[2,+,*], and Christine Froidevaux[4,+]

[1]Biotechnology Research Institute for Drug Discovery, National Institute of Advanced Industrial Science and Technology (AIST), Aomi, Tokyo 135-0064, Japan
[2]PRC, INRA, CNRS, Université François Rabelais-Tours, 37380, Nouzilly, France
[3]CNRS ; Université François-Rabelais de Tours, UMR 7292, 37032 Tours, France
[4]Laboratoire de Recherche en Informatique UMR CNRS 8623, Université Paris-Sud, Université Paris-Saclay, Orsay Cedex, 91405, France
[*]anne.poupon@inra.fr
[+]these authors contributed equally to this work

Listing of predicates

Background knowledge predicates

- **Predicate:** *molecule(X)*
  Typing: none
  Meaning: *X* is a molecule.

- **Predicate:** *simpleChemical(X)*
  Typing: none
  Meaning: *X* is a simple chemical.

- **Predicate:** *gene(X)*
  Typing: none
  Meaning: *X* is a gene.

- **Predicate:** *mrna(X)*
  Typing: none
  Meaning: *X* is a mRNA.

- **Predicate:** *protein(X)*
  Typing: none
  Meaning: *X* is a protein.

- **Predicate:** *acetylProtein(X)*
  Typing: none
  Meaning: *X* is an acetylated protein.

- **Predicate:** *phosphoProtein(X)*
  Typing: none
  Meaning: *X* is a phosphorylated protein.

- **Predicate:** *complex(X)*
  Typing: none
  Meaning: *X* is a complex.

- **Predicate:** *relation(R)*
  Typing: $R \in \{transcription, translation\}$
  Meaning: *R* is a relation (transcription or translation).

- **Predicate:** *antibody(A)*
  Typing: none
  Meaning: *A* is an antibody.

- **Predicate:** *panAntibody(A)*
  Typing: none
  Meaning: *A* is a pan-antibody.

- **Predicate:** *antagonist(I)*
  Typing: none
  Meaning: *I* is an antagonist.

- **Predicate:** *inhibitor(I)*
  Typing: none
  Meaning: *I* is an inhibitor.

- **Predicate:** *sirna(I)*
  Typing: none
  Meaning: *I* is a siRNA.

- **Predicate:** *antibodyAgainst(A, X)*
  Typing: *A*: antibody; *X*: protein
  Meaning: Antibody *A* targets protein *X*.

- **Predicate:** *panAntibodyAgainst(A, X)*
  Typing: *A*: pan-antibody; *X*: protein
  Meaning: Pan-antibody *A* targets protein *X* and all its modified forms.

- **Predicate:** *antagonistAgainst(I, X)*
  Typing: *I*: siRNA; *X*: protein
  Meaning: *I* is an antagonist of protein *X*.

- **Predicate:** *inhibitorAgainst(I, X)*
  Typing: *I*: inhibitor; *X*: molecule
  Meaning: Inhibitor *I* targets molecule *X*.

- **Predicate:** *relationInhibitorAgainst(I, R)*
  Typing: *I*: inhibitor; *R*: relation
  Meaning: Inhibitor *I* targets relation *R*.

- **Predicate:** *sirnaAgainst(I, X)*
  Typing: *I*: siRNA; *X*: protein
  Meaning: siRNA *I* targets protein *X*.

- **Predicate:** *notModified(X)*
  Typing: *X*: molecule
  Meaning: Molecule *X* has no modified form.

- **Predicate:** *modifiedForm(X, Y)*
  Typing: *X*: molecule; *Y*: molecule
  Meaning: Molecule *X* is a modified form of molecule *Y*.

- **Predicate:** *radioLabeledForm(X, Y)*
  Typing: *X*: molecule; *Y*: molecule
  Meaning: Molecule *X* is a radio labeled form of molecule *Y*.

- **Predicate:** *acetylForm(X, Y, P)*
  Typing: *X*: molecule; *Y*: molecule
  Meaning: Molecule *X* is the acetylated form of molecule *Y* at position *P*.

- **Predicate:** *phosphoForm(X, Y, P)*
  Typing: X: molecule; Y: molecule
  Meaning: Molecule X is the phophorylated form of molecule Y at position P.

- **Predicate:** *transcribed(X, Y)*
  Typing: X: gene; Y: mRNA
  Meaning: Gene X is transcribed into mRNA Y.

- **Predicate:** *translated(X, Y)*
  Typing: X: mRNA; Y: protein
  Meaning: mRNA X is translated into protein Y.

- **Predicate:** *functionalEquivalent(X, Y)*
  Typing: X: molecule; Y: molecule
  Meaning: X is a functional equivalent of Y.

Network predicates

- **Predicate:** *localized(X, C, S)*
  Typing: X: molecule; C: compartment; $S \in \{hypothesis, confirmed, infirmed\}$
  Meaning: Molecule X is localized in compartment C, with status S.

- **Predicate:** *notlocalized(X, C, S)*
  Typing: X: molecule; C: compartment; $S \in \{hypothesis, confirmed, infirmed\}$
  Meaning: Molecule X is not localized in compartment C, with status S.

- **Predicate:** *association(S1, C, S)*
  Typing: $S1$: multiset of molecules; C: complex; $S \in \{hypothesis, confirmed, infirmed\}$
  Meaning: There is an association process that transforms the molecules of $S1$ into complex C, with a status S.

- **Predicate:** *dissociation(C, S1, S)*
  Typing: C: complex; $S1$: multiset of molecules; $S \in \{hypothesis, confirmed, infirmed\}$
  Meaning: There is a dissociation process that transforms C into the molecules of $S1$, with a status S.

- **Predicate:** *modulates(X, Y1, Y2, E, D, S)*
  Typing: X: molecule; $Y1$: molecule; $Y2$: molecule; $E \in \{increase, decrease, noeffect\}$; $D \in \{unknown, direct, indirect\}$; $S \in \{hypothesis, confirmed, infirmed\}$
  Meaning: Molecule X influences the reaction that transforms molecule $Y1$ into molecule $Y2$ with an effect E, a distance D, and a status S.

- **Predicate:** *catalyzes(X, S1, S2, S)*
  Typing: X: molecule; $S1$: multiset of molecules; $S2$: multiset of molecules; $S \in \{hypothesis, confirmed, infirmed\}$
  Meaning: Molecule X catalyzes the reaction that transforms the molecules of $S1$ into the molecules of $S2$, with a status S.

- **Predicate:** *modulatesAssociation(X, S1, C, E, D, S)*
  Typing: X: molecule; $S1$: multiset of molecules; C: complex; $E \in \{increase, decrease, noeffect\}$; $D \in \{unknown, direct, indirect\}$; $S \in \{hypothesis, confirmed, infirmed\}$
  Meaning: Molecule X influences the association process that transforms molecules of $S1$ into complex C with an effect E, a distance D, and a status S.

- **Predicate:** *modulatesDissociation(X, C, S1, E, D, S)*
  Typing: X: molecule; C: complex; $S1$: multiset of molecules; $E \in \{increase, decrease, noeffect\}$; $D \in \{unknown, direct, indirect\}$; $S \in \{hypothesis, confirmed, infirmed\}$
  Meaning: Molecule X influences the dissociation process that transforms complex C into the molecules of $S1$ with an effect E, a distance D, and a status S.

- **Predicate:** *modulatesTranscription(X, Y, Z, E, D, S)*
  Typing: X: molecule; Y: gene; Z: mRNA; $E \in \{increase, decrease, noeffect\}$; $D \in \{unknown, direct, indirect\}$; $S \in \{hypothesis, confirmed, infirmed\}$
  Meaning: Molecule X influences the transcription of gene Y into mRNA Z with an effect E, a distance D, and a status S.

- **Predicate:** *modulatesTranslation(X, Y, Z, E, D, S)*
  Typing: X: molecule; Y: mRNA; Z: protein; $E \in \{increase, decrease, noeffect\}$; $D \in \{unknown, direct, indirect\}$; $S \in \{hypothesis, confirmed, infirmed\}$
  Meaning: Molecule X influences the translation of mRNA Y into protein Z with an effect E, a distance D, and a status S.

Experimental result predicates

- **Short name:** EA
  Full name: Enzymatic Assay
  Predicate: *ea(X,S1,S2,E)*
  Typing: X: molecule; *S1*: multiset of molecules; *S2*: multiset of molecules; $E \in \{increase, decrease, noeffect\}$
  Meaning: An enzymatic assay shows effect E of molecule X on the reaction that transforms molecules of *S1* into molecules of *S2*.

- **Short name:** ACPEA
  Full name: Enzymatic assay with an antagonist
  Predicate: *acpea(X, S1, S2, I, E)*
  Typing: X: molecule; *S1*: multiset of molecules; *S2*: multiset of molecules; I: antagonist; $E \in \{increase, decrease, noeffect\}$
  Meaning: An enzymatic assay shows effect E of the antagonist I on the reaction that transforms molecules of *S1* into molecules of *S2* and that is catalyzed by molecule X.

- **Short name:** ICPEA
  Full name: Enzymatic assay with an inhibitor
  Predicate: *icpea(X, S1, S2, I, E)*
  Typing: X: molecule; *S1*: multiset of molecules; *S2*: multiset of molecules; I: inhibitor; $E \in \{increase, decrease, noeffect\}$
  Meaning: An enzymatic assay shows effect E of the inhibitor I on the reaction that transforms molecules of *S1* into molecules of *S2* and that is catalyzed by molecule X.

- **Short name:** SCPEA
  Full name: Enzymatic assay with a siRNA
  Predicate: *scpea(X, S1, S2, I, E)*
  Typing: X: molecule; *S1*: multiset of molecules; *S2*: multiset of molecules; I: siRNA; $E \in \{increase, decrease, noeffect\}$
  Meaning: An enzymatic assay shows effect E of siRNA I on the reaction that transforms molecules of *S1* into molecules of *S2* and that is catalyzed by molecule X.

- **Short name:** PA
  Full name: Phosphorylation assay with an antibody
  Predicate: *pa(X, Y, A, E)*
  Typing: X: molecule; Y: protein; A: antibody; $E \in \{increase, decrease, noeffect\}$
  Meaning: A phosphorylation assay shows effect E of molecule X on the reaction of phosphorylation that transforms protein Y into its phosphorylated form detected by means of antibody A.

- **Short name:** PRA
  Full name: Phosphorylation assay with a radio-labeled form
  Predicate: *pra(X, Y, A, E)*
  Typing: X: molecule; Y: protein; A: radio-labeled molecule; $E \in \{increase, decrease, noeffect\}$
  Meaning: A phosphorylation assay shows an effect E of molecule X on the reaction of phosphorylation that transforms protein Y into its phosphorylated form detected by means of radio-labeled form A.

- **Short name:** ACPPA
  Full name: Phosphorylation assay with an antibody and an antagonist
  Predicate: *acppa(X, Y, A, I, E)*
  Typing: X: molecule; Y: protein; A: antibody; I: antagonist; $E \in \{increase, decrease, noeffect\}$
  Meaning: A phosphorylation assay shows effect E of antagonist I on the reaction of phosphorylation that transforms protein Y into its phosphorylated form detected by means of antibody A and that is influenced by molecule X.

- **Short name:** ICPPA
  Full name: Phosphorylation assay with an antibody and an inhibitor
  Predicate: *icppa(X, Y, A, I, E)*

Typing: X: molecule; Y: protein; A: antibody; I: inhibitor; $E \in \{increase, decrease, noeffect\}$
Meaning: A phosphorylation assay shows effect E of inhibitor I on the reaction of phosphorylation that transforms protein Y into its phosphorylated form detected by means of antibody A and that is influenced by molecule X.

- **Short name:** ICPPRA
Full name: Phosphorylation assay with a radio-labeled form and an inhibitor
Predicate: *icppra(X, Y, A, I, E)*
Typing: X: molecule; Y: protein; A: radio-labeled molecule; I: inhibitor; $E \in \{increase, decrease, noeffect\}$
Meaning: A phosphorylation assay shows effect E of inhibitor I on the reaction of phosphorylation that transforms protein Y into its phosphorylated form detected by means of radio-labeled form A and that is influenced by molecule X.

- **Short name:** SCPPA
Full name: Phosphorylation assay with an antibody and a siRNA
Predicate: *scppa(X, Y, A, I, E)*
Typing: X: molecule; Y: protein; A: antibody; I: siRNA; $E \in \{increase, decrease, noeffect\}$
Meaning: A phosphorylation assay shows effect E of siRNA I on the reaction of phosphorylation that transforms protein Y into its phosphorylated form detected by means of antibody A and that is influenced by molecule X.

- **Short name:** ELISA
Full name: Enzyme-linked immunosorbent assay
Predicate: *elisa(X, A, E)*
Typing: X: molecule; A: antibody; $E \in \{increase, decrease, noeffect\}$
Meaning: An enzyme-linked immunosorbent assay shows the effect E of molecule X on the quantity of the molecule that is detected by means of antibody A.

- **Short name:** ICELISA
Full name: Enzyme-linked immunosorbent assay with an inhibitor
Predicate: *icelisa(X, A, I, E)*
Typing: X: molecule; A: antibody; I: inhibitor; $E \in \{increase, decrease, noeffect\}$
Meaning: An enzyme-linked immunosorbent assay shows the effect E of inhibitor I on the quantity of the molecule that is detected by means of antibody A and that is influenced by molecule X.

- **Short name:** SCELISA
Full name: Enzyme-linked immunosorbent assay with a siRNA
Predicate: *scelisa(X, A, I, E)*
Typing: X: molecule; A: antibody; I: siRNA; $E \in \{increase, decrease, noeffect\}$
Meaning: An enzyme-linked immunosorbent assay shows the effect E of siRNA I on the quantity of the molecule that is detected by means of antibody A and that is influenced by molecule X.

- **Short name:** RIA
Full name: Radio-immunology assay
Predicate: *ria(X, A, E)*
Typing: X: molecule; A: antibody; $E \in \{increase, decrease, noeffect\}$
Meaning: A radio-immunology assay shows an effect E of molecule X on the quantity of the molecule detected by means of antibody A.

- **Short name:** ICRIA
Full name: Radio-immunology assay with an inhibitor
Predicate: *ria(X, A, I, E)*
Typing: X: molecule; A: antibody; I: inhibitor; $E \in \{increase, decrease, noeffect\}$
Meaning: A radio-immunology assay shows an effect E of inhibitor I on the quantity of the molecule detected by means of antibody A and that is influenced by molecule X.

- **Short name:** WB
Full name: Western Blot with an antibody
Predicate: *wb(X, A, E)*
Typing: X: molecule; A: antibody; $E \in \{increase, decrease, noeffect\}$
Meaning: A Western blot shows an effect E of molecule X on the quantity of the molecule detected by means of pan-antibody A.

- **Short name:** ICWB
Full name: Western Blot with an antibody and an inhibitor
Predicate: *icWb(X, A, I, E)*
Typing: X: molecule; A: antibody; I: inhibitor; $E \in \{increase, decrease, noeffect\}$
Meaning: A Western blot shows an effect E of inhibitor I on the quantity of the molecule detected by means of pan-antibody A and that is influenced by molecule X.

- **Short name:** RIWB
Full name: Western Blot with an antibody and an inhibitor of relation (transcription or translation)
Predicate: *riWb(X, A, I, E)*
Typing: X: molecule; A: pan-antibody; I: inhibitor; $E \in \{increase, decrease, noeffect\}$
Meaning: A Western blot shows an effect E of inhibitor of relation (transcription or translation) I on the quantity of the molecule detected by means of pan-antibody A and that is influenced by molecule X.

- **Short name:** QRTPCR
Full name: Quantitative reverse transcription polymerase chain reaction assay
Predicate: *qrtpcr(X, Y, E)*
Typing: X: molecule; Y: mRNA; $E \in \{increase, decrease, noeffect\}$
Meaning: A quantitative reverse transcription polymerase chain reaction assay shows an effect E of molecule X on the transcription of a given gene into mRNA Y.

- **Short name:** ICQRTPCR
Full name: Quantitative reverse transcription polymerase chain reaction assay with an inhibitor
Predicate: *icqrtpcr(X, Y, I, E)*
Typing: X: molecule; Y: mRNA; I: inhibitor; $E \in \{increase, decrease, noeffect\}$
Meaning: A quantitative reverse transcription polymerase chain reaction assay shows an effect E of inhibitor Y on the transcription of a given gene into mRNA Y that is influenced by molecule X.

- **Short name:** IP
Full name: Immunoprecipitation assay
Predicate: *ip(X,S)*
Typing: X: molecule; S: multiset of molecules
Meaning: An immunoprecipitation assay shows that molecules of S co-immunoprecipate with molecule X.

- **Short name:** IPD
Full name: Immunoprecipitation assay with an additional molecule
Predicate: *ipd(X, Y, S, E)*
Typing: X: molecule; Y: molecule; S: multiset of molecules; $E \in \{increase, decrease, noeffect\}$
Meaning: An immunoprecipitation assay shows that molecule X has an effect E on the co-immunoprecipitation of the molecules of $S1$ with molecule Y.

- **Short name:** GSTPULLDOWN
Full name: Gluthatione-S-Transferase pull down assay
Predicate: *gstPulldown(X, S)*
Typing: X: molecule; S: multiset of molecules
Meaning: A Gluthatione-S-Transferase pulldown assay shows that molecule X physically interacts with the molecules of S.

- **Short name:** BDPULLDOWN
Full name: Binding domain pull down assay
Predicate: *bdPulldown(X, S)*
Typing: X: molecule; S: multiset of molecules
Meaning: A binding domain pulldown assay shows that molecule X physically interacts with the molecules of S.

- **Short name:** FRET
Full name: Fluorescence resonance energy transfert assay
Predicate: *fret(X, Y)*
Typing: X: molecule; Y: molecule
Meaning: A fluorescence resonance energy transfer assay shows that molecule X physically interacts with molecule Y.

- **Short name:** 3D
  Full name: Cristallography assay
  Predicate: *3d(S)*
  Typing: S: multiset of molecules
  Meaning: A cristallography assay shows that molecules of S interact physically together.

- **Short name:** FLUO
  Full name: Localization by fluorescence
  Predicate: *fluo(A, C, E)*
  Typing: A: antibody; C: compartment; $E \in : \{positive, negative\}$
  Meaning: A fluorescence assay about presence in compartment C of the molecule detected by means of antibody A shows an E result.

- **Short name:** IHC
  Full name: Localization by immunohistochemistry
  Predicate: *ihc(A, C, E)*
  Typing: A: antibody; C: compartment; $E \in : \{positive, negative\}$
  Meaning: An immunohistochemistry assay about presence in compartment C of the molecule detected by means of antibody A shows an E result.

Rule generalization

The two following main classes of disruptors can be distinguished:

- specific disruptors, such as inhibitors and antagonists, that prevent the activation of a particular molecule, by inhibiting the catalyzer of the activation process for example. As a result, this type of inhibitors is specific to a particular form of a molecule.
- non specific disruptors, such as siRNAs or knock-downs, that target all forms of a given molecule either by deleting the gene (knock down) or by greatly reducing its expression (siRNA).

These two classes can be formalized by the two predicates *specificDisruptor* and *unspecificDisruptor*, and the ontological relation between a particular disruptor and the class it belongs to by a simple rule. For example, the following ontological rule specifies the fact that any inhibitor is a specific disruptor:

$$\text{IF } \mathit{inhibitor}(I,X) \text{ THEN } \mathit{specificDisruptor}(I,X) \tag{R1}$$

For each experiment type, the measures are realized either directly or by means of a detector (e.g. an antibody or a radio labeled form). Again, two classes of detectors can be distinguished, based on their specificity: an antibody specific to a given phosphorylation of a protein targets only forms of the protein that contain this specific phosphorylation, whereas a pan antibody targets all post-translational modifications of this same protein. As for the disruptors, this classification can be formalized by introducing two predicates: *specificDetector* and *unspecificDetector*, and rules analogous to rule (R1).

The classification of the different detectors and disruptors into two classes (specific and unspecific), and the classification of the experiment types according to different criteria can be taken into account in order to generalize interpretative rules further. For example, the *pa* and the *pra* experiment types are both simple phosphorylation assays that use specific detectors (an antibody in the case of *pa*, and a radio labeled form in the case of *pra*), and that bring into play the same processes. Hence, these two experiment types can be gathered into one more general experiment type *simplePa*, and the experimental results obtained when realizing one or the other experiment can be formalized by the predicate $\mathit{simplePa}(X,Y,D,E)$, considering the following ontological rules:

$$\text{IF } \mathit{pa}(X,Y,D,E) \text{ THEN } \mathit{simplePa}(X,Y,D,E) \tag{R2}$$

and

$$\text{IF } \mathit{pra}(X,Y,D,E) \text{ THEN } \mathit{simplePa}(X,Y,D,E) \tag{R3}$$

Hence, the rules allowing to interpret results from these two experiment types can be generalized into only one interpretative rule :

$$\begin{aligned}&\text{IF } \mathit{simplePa}(X,Y,D,E)\\ &\text{and } \mathit{specificDetector}(D,Y^a) \text{ and } \mathit{modifiedForm}(Y^a,Y)\\ &\text{THEN } \mathit{modulates}(X,Y,Y^a,E,\mathit{unknown},\mathit{confirmed})\end{aligned} \tag{R4}$$

Listing of rules

Ontological rules

$$\begin{aligned}&\text{IF } \mathit{protein}(X)\\ &\text{THEN } \mathit{molecule}(X)\end{aligned} \tag{R5}$$

$$\begin{aligned}&\text{IF } \mathit{simplechemical}(X)\\ &\text{THEN } \mathit{molecule}(X)\end{aligned} \tag{R6}$$

$$\begin{aligned}&\text{IF } \mathit{mrna}(X)\\ &\text{THEN } \mathit{molecule}(X)\end{aligned} \tag{R7}$$

$$\begin{aligned}&\text{IF } \mathit{gene}(X)\\ &\text{THEN } \mathit{molecule}(X)\end{aligned} \tag{R8}$$

$$\begin{aligned}&\text{IF } \mathit{phosphoprotein}(X)\\ &\text{THEN } \mathit{protein}(X)\end{aligned} \tag{R9}$$

$$\begin{aligned}&\text{IF } \mathit{acetylprotein}(X)\\ &\text{THEN } \mathit{protein}(X)\end{aligned} \tag{R10}$$

$$\begin{aligned}&\text{IF } \mathit{panantibody}(X)\\ &\text{THEN } \mathit{antibody}(X)\end{aligned} \tag{R11}$$

$$\begin{aligned}&\text{IF } \mathit{antibodyagainst}(A,X)\\ &\text{THEN } \mathit{specificdetector}(A,X)\end{aligned} \tag{R12}$$

$$\begin{aligned}&\text{IF } \mathit{radiolabelledform}(R,X)\\ &\text{THEN } \mathit{specificdetector}(R,X)\end{aligned} \tag{R13}$$

$$\begin{aligned}&\text{IF } \mathit{panantibodyagainst}(A,X)\\ &\text{THEN } \mathit{unspecificdetector}(A,X)\end{aligned} \tag{R14}$$

$$\begin{aligned}&\text{IF } \mathit{inhibitoragainst}(I,X)\\ &\text{THEN } \mathit{specificdisruptor}(I,X)\end{aligned} \tag{R15}$$

$$\begin{aligned}&\text{IF } \mathit{antagonist}(A,X)\\ &\text{THEN } \mathit{specificdisruptor}(A,X)\end{aligned} \tag{R16}$$

$$\begin{aligned}&\text{IF } \mathit{sirnaagainst}(S,X)\\ \text{THEN }&\mathit{unspecificdisruptor}(S,X)\end{aligned} \tag{R17}$$

$$\begin{aligned}&\text{IF } \mathit{ko}(K,X)\\ \text{THEN }&\mathit{unspecificdisruptor}(K,X)\end{aligned} \tag{R18}$$

$$\begin{aligned}&\text{IF } \mathit{agonist}(X,Y)\\ \text{THEN }&\mathit{functionalequivalent}(X,Y)\end{aligned} \tag{R19}$$

$$\begin{aligned}&\text{IF } \mathit{specificdetector}(D,X)\\ \text{THEN }&\mathit{detector}(D,X)\end{aligned} \tag{R20}$$

$$\begin{aligned}&\text{IF } \mathit{unspecificdetector}(D,X)\\ \text{THEN }&\mathit{detector}(D,X)\end{aligned} \tag{R21}$$

$$\begin{aligned}&\text{IF } \mathit{specificdisruptor}(P,X)\\ \text{THEN }&\mathit{disruptor}(P,X)\end{aligned} \tag{R22}$$

$$\begin{aligned}&\text{IF } \mathit{unspecificdisruptor}(P,X)\\ \text{THEN }&\mathit{disruptor}(P,X)\end{aligned} \tag{R23}$$

$$\begin{aligned}&\text{IF } \mathit{pa}(X,Y,D,E)\\ \text{THEN }&\mathit{simplepa}(X,Y,D,E)\end{aligned} \tag{R24}$$

$$\begin{aligned}&\text{IF } \mathit{pra}(X,Y,D,E)\\ \text{THEN }&\mathit{simplepa}(X,Y,D,E)\end{aligned} \tag{R25}$$

$$\begin{aligned}&\text{IF } \mathit{aa}(X,Y,D,E)\\ \text{THEN }&\mathit{simplepa}(X,Y,D,E)\end{aligned} \tag{R26}$$

$$\begin{aligned}&\text{IF } \mathit{elisa}(X,D,E)\\ \text{THEN }&\mathit{simpleelisa}(X,D,E)\end{aligned} \tag{R27}$$

$$\begin{aligned}&\text{IF } \mathit{ria}(X,D,E)\\ \text{THEN }&\mathit{simpleria}(X,D,E)\end{aligned} \tag{R28}$$

$$\begin{aligned}&\text{IF } \mathit{wb}(X,D,E)\\ \text{THEN }&\mathit{simplewb}(X,D,E)\end{aligned} \tag{R29}$$

$$\begin{aligned}&\text{IF } \mathit{ea}(X,Sr,Sp,\mathit{1})\\ \text{THEN }&\mathit{simpleea}(X,Sr,Sp,\mathit{1})\end{aligned} \tag{R30}$$

$$\begin{array}{l}\text{IF } icppa(X,Z,P,D,E) \\ \text{THEN } complexpa(X,Z,P,D,E)\end{array} \tag{R31}$$

$$\begin{array}{l}\text{IF } icppra(X,Z,P,D,E) \\ \text{THEN } complexpa(X,Z,P,D,E)\end{array} \tag{R32}$$

$$\begin{array}{l}\text{IF } scppa(X,Z,P,D,E) \\ \text{THEN } complexpa(X,Z,P,D,E)\end{array} \tag{R33}$$

$$\begin{array}{l}\text{IF } koppa(X,Z,P,D,E) \\ \text{THEN } complexpa(X,Z,P,D,E)\end{array} \tag{R34}$$

$$\begin{array}{l}\text{IF } icelisa(X,P,D,E) \\ \text{THEN } complexelisa(X,P,D,E)\end{array} \tag{R35}$$

$$\begin{array}{l}\text{IF } icria(X,P,D,E) \\ \text{THEN } complexria(X,P,D,E)\end{array} \tag{R36}$$

$$\begin{array}{l}\text{IF } icwb(X,P,D,E) \\ \text{THEN } complexwb(X,P,D,E)\end{array} \tag{R37}$$

$$\begin{array}{l}\text{IF } qrtpcr(X,Y,E) \\ \text{THEN } simpleqrtpcr(X,Y,E)\end{array} \tag{R38}$$

$$\begin{array}{l}\text{IF } icqrtpcr(X,Y,P,E) \\ \text{THEN } complexqrtpcr(X,Y,P,E)\end{array} \tag{R39}$$

$$\begin{array}{l}\text{IF } icpea(X,Sr,Sp,P,E) \\ \text{THEN } complexea(X,Sr,Sp,P,E)\end{array} \tag{R40}$$

$$\begin{array}{l}\text{IF } acpea(X,Sr,Sp,P,E) \\ \text{THEN } complexea(X,Sr,Sp,P,E)\end{array} \tag{R41}$$

$$\begin{array}{l}\text{IF } fluo(D,C,E) \\ \text{THEN } detectionexp(D,C,E)\end{array} \tag{R42}$$

$$\begin{array}{l}\text{IF } ihc(D,C,E) \\ \text{THEN } detectionexp(D,C,E)\end{array} \tag{R43}$$

$$\begin{array}{l}\text{IF } bdpulldown(X,S) \\ \text{THEN } onetomanyinteractionexp(X,S)\end{array} \tag{R44}$$

$$\begin{aligned}&\text{IF } \mathit{gstpulldown}(X,S)\\ &\text{THEN } \mathit{onetomanyinteractionexp}(X,S)\end{aligned} \tag{R45}$$

$$\begin{aligned}&\text{IF } \mathit{ip}(X,S)\\ &\text{THEN } \mathit{onetomanyinteractionexp}(X,S)\end{aligned} \tag{R46}$$

$$\begin{aligned}&\text{IF } \mathit{fret}(X,Y)\\ &\text{THEN } \mathit{onetooneinteractionexp}(X,Y)\end{aligned} \tag{R47}$$

$$\begin{aligned}&\text{IF } \mathit{threed}(S)\\ &\text{THEN } \mathit{manyinteractionexp}(S)\end{aligned} \tag{R48}$$

$$\begin{aligned}&\text{IF } \mathit{ipd}(X,Y,S,E)\\ &\text{THEN } \mathit{effectononetomanyinteractionexp}(X,Y,S,E)\end{aligned} \tag{R49}$$

Interpretative rules

In the following rules, the *increase* and *decrease* constants are encoded by 1 and -1, respectively. This encoding enables the use of the multiplcation operator ($*$) between effects, and therefore to write a lower number of rules.

$$\begin{aligned}&\text{IF } \mathit{simplepa}(Xa,Y,A,E)\\ &\text{and } specificdetector(A,Ya)\\ &\text{and } modifiedform(Ya,Y)\\ &\text{THEN } \mathit{modulates}(Xa,Y,Ya,E,\mathit{unknown},\mathit{confirmed})\end{aligned} \tag{R50}$$

$$\begin{aligned}&\text{IF } \mathit{simpleelisa}(Xa,D,E)\\ &\text{and } specificdetector(D,Ya)\\ &\text{and } modifiedform(Ya,Y)\\ &\text{THEN } \mathit{modulates}(Xa,Y,Ya,E,\mathit{unknown},\mathit{hypothesis})\end{aligned} \tag{R51}$$

$$\begin{aligned}&\text{IF } \mathit{simpleria}(Xa,D,E)\\ &\text{and } specificdetector(D,Ya)\\ &\text{and } modifiedform(Ya,Y)\\ &\text{THEN } \mathit{modulates}(Xa,Y,Ya,E,\mathit{unknown},\mathit{hypothesis})\end{aligned} \tag{R52}$$

$$\begin{aligned}&\text{IF } \mathit{simpleea}(Xa,Sr,Sp,\mathit{1})\\ &\text{THEN } \mathit{catalyzes}(Xa,Sr,Sp,\mathit{confirmed})\end{aligned} \tag{R53}$$

$$\begin{aligned}&\text{IF } \mathit{complexpa}(Xa,Z,P,D,\mathit{E1})\\ &\text{and } modulates(Xa,Z,Za,E2,unknown,confirmed)\\ &\text{and } specificdetector(D,Za)\\ &\text{and } specificdisruptor(P,Ya)\\ &\text{and } modifiedform(Ya,Y)\\ &\text{and } notmodified(Xa)\\ &\text{THEN } \mathit{modulates}(Ya,Z,Za,\mathit{-E1},\mathit{unknown},\mathit{confirmed})\\ &\text{and } \mathit{modulates}(Xa,Y,Ya,\mathit{-E1*E2},\mathit{unknown},\mathit{hypothesis})\end{aligned} \tag{R54}$$

$$
\begin{aligned}
&\text{IF } complexpa(Xa, Z, P, D, E1)\\
&\text{and } modulates(Xa, Z, Za, E2, unknown, confirmed)\\
&\text{and } specificdetector(D, Za)\\
&\text{and } specificdisruptor(P, Ya)\\
&\text{and } modifiedform(Ya, Y)\\
&\text{and } modifiedform(Xa, X)\\
&\text{THEN } modulates(Ya, Z, Za, \text{-}E1, unknown, confirmed)\\
&\text{and } modulates(Xa, Y, Ya, \text{-}E1*E2, unknown, hypothesis)\\
&\text{and } modulates(Ya, X, Xa, \text{-}E1*E2, unknown, hypothesis)
\end{aligned} \tag{R55}
$$

$$
\begin{aligned}
&\text{IF } complexpa(Xa, Z, P, D, E1)\\
&\text{and } modulates(Xa, Z, Za, E2, unknown, confirmed)\\
&\text{and } specificdetector(D, Za)\\
&\text{and } unspecificdisruptor(P, Ya)\\
&\text{and } modifiedform(Ya, Y)\\
&\text{and } notmodified(Xa)\\
&\text{THEN } modulates(Ya, Z, Za, \text{-}E1, unknown, hypothesis)\\
&\text{and } modulates(Xa, Y, Ya, \text{-}E1*E2, unknown, hypothesis)
\end{aligned} \tag{R56}
$$

$$
\begin{aligned}
&\text{IF } complexpa(Xa, Z, P, D, E1)\\
&\text{and } modulates(Xa, Z, Za, E2, unknown, confirmed)\\
&\text{and } specificdetector(D, Za)\\
&\text{and } unspecificdisruptor(P, Ya)\\
&\text{and } modifiedform(Ya, Y)\\
&\text{and } modifiedform(Xa, X)\\
&\text{THEN } modulates(Ya, Z, Za, \text{-}E1, unknown, hypothesis)\\
&\text{and } modulates(Xa, Y, Ya, \text{-}E1*E2, unknown, hypothesis)\\
&\text{and } modulates(Ya, X, Xa, \text{-}E1*E2, unknown, hypothesis)
\end{aligned} \tag{R57}
$$

$$
\begin{aligned}
&\text{IF } complexelisa(Xa, P, D, E1)\\
&\text{and } modulates(Xa, Z, Za, E2, unknown, confirmed)\\
&\text{and } specificdetector(D, Za)\\
&\text{and } disruptor(P, Ya)\\
&\text{and } modifiedform(Ya, Y)\\
&\text{and } notmodified(Xa)\\
&\text{THEN } modulates(Ya, Z, Za, \text{-}E1, unknown, hypothesis)\\
&\text{and } modulates(Xa, Y, Ya, \text{-}E1*E2, unknown, hypothesis)
\end{aligned} \tag{R58}
$$

$$
\begin{aligned}
&\text{IF } complexelisa(Xa,P,D,E1)\\
&\text{and } modulates(Xa,Z,Za,E2,unknown,confirmed)\\
&\text{and } specificdetector(D,Za)\\
&\text{and } disruptor(P,Ya)\\
&\text{and } modifiedform(Ya,Y)\\
&\text{and } modifiedform(Xa,X)\\
&\text{THEN } modulates(Ya,Z,Za,\text{-}E1,unknown,hypothesis)\\
&\text{and } modulates(Xa,Y,Ya,\text{-}E1{*}E2,unknown,hypothesis)\\
&\text{and } modulates(Ya,X,Xa,\text{-}E1{*}E2,unknown,hypothesis)
\end{aligned}
\qquad \text{(R59)}
$$

$$
\begin{aligned}
&\text{IF } complexria(Xa,P,D,E1)\\
&\text{and } modulates(Xa,Z,Za,E2,unknown,confirmed)\\
&\text{and } specificdetector(D,Za)\\
&\text{and } disruptor(P,Ya)\\
&\text{and } modifiedform(Ya,Y)\\
&\text{and } notmodified(Xa)\\
&\text{THEN } modulates(Ya,Z,Za,\text{-}E1,unknown,hypothesis)\\
&\text{and } modulates(Xa,Y,Ya,\text{-}E1{*}E2,unknown,hypothesis)
\end{aligned}
\qquad \text{(R60)}
$$

$$
\begin{aligned}
&\text{IF } complexria(Xa,P,D,E1)\\
&\text{and } modulates(Xa,Z,Za,E2,unknown,confirmed)\\
&\text{and } specificdetector(D,Za)\\
&\text{and } disruptor(P,Ya)\\
&\text{and } modifiedform(Ya,Y)\\
&\text{and } modifiedform(Xa,X)\\
&\text{THEN } modulates(Ya,Z,Za,\text{-}E1,unknown,hypothesis)\\
&\text{and } modulates(Xa,Y,Ya,\text{-}E1{*}E2,unknown,hypothesis)\\
&\text{and } modulates(Ya,X,Xa,\text{-}E1{*}E2,unknown,hypothesis)
\end{aligned}
\qquad \text{(R61)}
$$

$$
\begin{aligned}
&\text{IF } simpleqrtpcr(Xa,Ya,E)\\
&\text{and } transcribed(Y,Ya)\\
&\text{THEN } modulatestranscription(Xa,Y,Ya,E,unknown,confirmed)
\end{aligned}
\qquad \text{(R62)}
$$

$$
\begin{aligned}
&\text{IF } complexqrtpcr(Xa,Za,P,E1)\\
&\text{and } modulatestranscription(Xa,Z,Za,E2,unknown,confirmed)\\
&\text{and } specificdisruptor(P,Ya)\\
&\text{and } modifiedform(Ya,Y)\\
&\text{and } notmodified(Xa)\\
&\text{THEN } modulates(Ya,Z,Za,\text{-}E1,unknown,confirmed)\\
&\text{and } modulates(Xa,Y,Ya,\text{-}E1{*}E2,unknown,hypothesis)
\end{aligned}
\qquad \text{(R63)}
$$

$$
\begin{aligned}
&\text{IF } complexqrtpcr(Xa, Za, P, E1)\\
&\text{and } modulates(Xa, Z, Za, E2, unknown, confirmed)\\
&\text{and } specificdisruptor(P, Ya)\\
&\text{and } modifiedform(Ya, Y)\\
&\text{and } modifiedform(Xa, X)\\
&\text{THEN } modulates(Ya, Z, Za, \text{-}E1, unknown, confirmed)\\
&\text{and } modulates(Xa, Y, Ya, \text{-}E1*E2, unknown, hypothesis)\\
&\text{and } modulates(Ya, X, Xa, \text{-}E1*E2, unknown, hypothesis)
\end{aligned}
\tag{R64}
$$

$$
\begin{aligned}
&\text{IF } complexqrtpcr(Xa, Za, P, E1)\\
&\text{and } modulates(Xa, Z, Za, E2, unknown, confirmed)\\
&\text{and } unspecificdisruptor(P, Ya)\\
&\text{and } modifiedform(Ya, Y)\\
&\text{and } notmodified(Xa)\\
&\text{THEN } modulates(Ya, Z, Za, \text{-}E1, unknown, hypothesis)\\
&\text{and } modulates(Xa, Y, Ya, \text{-}E1*E2, unknown, hypothesis)
\end{aligned}
\tag{R65}
$$

$$
\begin{aligned}
&\text{IF } complexqrtpcr(Xa, Za, P, E1)\\
&\text{and } modulates(Xa, Z, Za, E2, unknown, confirmed)\\
&\text{and } unspecificdisruptor(P, Ya)\\
&\text{and } modifiedform(Ya, Y)\\
&\text{and } modifiedform(Xa, X)\\
&\text{THEN } modulates(Ya, Z, Za, \text{-}E1, unknown, hypothesis)\\
&\text{and } modulates(Xa, Y, Ya, \text{-}E1*E2, unknown, hypothesis)\\
&\text{and } modulates(Ya, X, Xa, \text{-}E1*E2, unknown, hypothesis)
\end{aligned}
\tag{R66}
$$

$$
\begin{aligned}
&\text{IF } simplewb(Xa, D, E)\\
&\text{and } unspecificdetector(D, Y)\\
&\text{and } translated(Ym, Y)\\
&\text{THEN } modulatestranslation(Xa, Ym, Y, E, unknown, hypothesis)\\
&\text{and } modulatesdegradation(Xa, Y, \text{-}E, unknown, hypothesis)
\end{aligned}
\tag{R67}
$$

$$
\begin{aligned}
&\text{IF } simplewb(Xa, D, E)\\
&\text{and } unspecificdetector(D, Y)\\
&\text{and } translated(Ym, Y)\\
&\text{and } transcribed(Yg, Ym)\\
&\text{THEN } modulatestranscription(Xa, Yg, Ym, E, unknown, hypothesis)
\end{aligned}
\tag{R68}
$$

$$
\begin{aligned}
&\text{IF } simplewb(Xa, D, E)\\
&\text{and } specificdetector(D, Ya)\\
&\text{and } modifiedform(Ya, Y)\\
&\text{THEN } modulates(Xa, Y, Ya, E, unknown, hypothesis)
\end{aligned}
\tag{R69}
$$

$$
\begin{aligned}
&\text{IF } complexwb(Xa, D, P, E1)\\
&\quad\text{and } modulatestranslation(Xa, Zm, Z, E2, unknown, confirmed)\\
&\quad\text{and } unspecificdetector(D, Z)\\
&\quad\text{and } disruptor(P, Ya)\\
&\quad\text{and } modifiedform(Ya, Y)\\
&\quad\text{and } notmodified(Xa)\\
&\text{THEN } modulatestranslation(Ya, Zm, Z, \text{-}E1, unknown, hypothesis)\\
&\quad\text{and } modulates(Xa, Y, Ya, \text{-}E1{*}E2, unknown, hypothesis)
\end{aligned}
\tag{R70}
$$

$$
\begin{aligned}
&\text{IF } complexwb(Xa, D, P, E1)\\
&\quad\text{and } modulatestranscription(Xa, Zg, Zm, E2, unknown, confirmed)\\
&\quad\text{and } translated(Zm, Z)\\
&\quad\text{and } unspecificdetector(D, Z)\\
&\quad\text{and } disruptor(P, Ya)\\
&\quad\text{and } modifiedform(Ya, Y)\\
&\quad\text{and } notmodified(Xa)\\
&\text{THEN } modulatestranscription(Ya, Zg, Zm, \text{-}E1, unknown, hypothesis)\\
&\quad\text{and } modulates(Xa, Y, Ya, \text{-}E1{*}E2, unknown, hypothesis)
\end{aligned}
\tag{R71}
$$

$$
\begin{aligned}
&\text{IF } complexwb(Xa, D, P, E1)\\
&\quad\text{and } modulatesdegradation(Xa, Z, E2, unknown, confirmed)\\
&\quad\text{and } unspecificdetector(D, Z)\\
&\quad\text{and } disruptor(P, Ya)\\
&\quad\text{and } modifiedform(Ya, Y)\\
&\quad\text{and } notmodified(Xa)\\
&\text{THEN } modulatesdegradation(Ya, Z, \text{-}E1, unknown, hypothesis)\\
&\quad\text{and } modulates(Xa, Y, Ya, \text{-}E1{*}E2, unknown, hypothesis)
\end{aligned}
\tag{R72}
$$

$$
\begin{aligned}
&\text{IF } complexwb(Xa, D, P, E1)\\
&\quad\text{and } modulatestranslation(Xa, Zm, Z, E2, unknown, confirmed)\\
&\quad\text{and } unspecificdetector(D, Z)\\
&\quad\text{and } disruptor(P, Ya)\\
&\quad\text{and } modifiedform(Ya, Y)\\
&\quad\text{and } modifiedform(Xa, X)\\
&\text{THEN } modulatestranslation(Ya, Zm, Z, \text{-}E1, unknown, hypothesis)\\
&\quad\text{and } modulates(Ya, X, Xa, \text{-}E1{*}E2, unknown, hypothesis)
\end{aligned}
\tag{R73}
$$

IF $complexwb(Xa, D, P, E1)$
and $modulatestranscription(Xa, Zg, Zm, E2, unknown, confirmed)$
and $translated(Zm, Z)$
and $unspecificdetector(D, Z)$
and $disruptor(P, Ya)$ (R74)
and $modifiedform(Ya, Y)$
and $modifiedform(Xa, X)$
THEN $modulatestranscription(Ya, Zg, Zm, \text{-}E1, unknown, hypothesis)$
and $modulates(Ya, X, Xa, \text{-}E1*E2, unknown, hypothesis)$

IF $complexwb(Xa, D, P, E1)$
and $modulatesdegradation(Xa, Z, E2, unknown, confirmed)$
and $unspecificdetector(D, Z)$
and $disruptor(P, Ya)$
and $modifiedform(Ya, Y)$ (R75)
and $modifiedform(Xa, X)$
THEN $modulatesdegradation(Ya, Z, \text{-}E1, unknown, hypothesis)$
and $modulates(Ya, X, Xa, \text{-}E1*E2, unknown, hypothesis)$

IF $complexwb(Xa, P, D, E1)$
and $modulates(Xa, Z, Za, E2, unknown, confirmed)$
and $specificdetector(D, Za)$
and $disruptor(P, Ya)$ (R76)
and $modifiedform(Ya, Y)$
and $notmodified(Xa)$
THEN $modulates(Ya, Z, Za, \text{-}E1, unknown, hypothesis)$
and $modulates(Xa, Y, Ya, \text{-}E1*E2, unknown, hypothesis)$

IF $complexwb(Xa, P, D, E1)$
and $modulates(Xa, Z, Za, E2, unknown, confirmed)$
and $specificdetector(D, Za)$
and $disruptor(P, Ya)$
and $modifiedform(Ya, Y)$ (R77)
and $modifiedform(Xa, X)$
THEN $modulates(Ya, Z, Za, \text{-}E1, unknown, hypothesis)$
and $modulates(Xa, Y, Ya, \text{-}E1*E2, unknown, hypothesis)$
and $modulates(Ya, X, Xa, \text{-}E1*E2, unknown, hypothesis)$

IF $complexea(Xa, Sr, Sp, P, E)$
and $catalyzes(Xa, Sr, Sp, confirmed)$
and $disruptor(P, Ya)$ (R78)
and $modifiedform(Xa, X)$
THEN $modulates(Ya, X, Xa, \text{-}E, unknown, hypothesis)$

$$\begin{aligned}&\text{IF } detectionexp(D,C,1)\\&\text{and } specificdetector(D,X)\\&\text{THEN } localized(X,C,confirmed)\end{aligned} \tag{R79}$$

$$\begin{aligned}&\text{IF } detectionexp(D,C,\text{-}1)\\&\text{and } specificdetector(D,X)\\&\text{THEN } notlocalized(X,C,confirmed)\end{aligned} \tag{R80}$$

$$\begin{aligned}&\text{IF } detectionexp(D,C,1)\\&\text{and } unspecificdetector(D,X)\\&\text{THEN } localized(X,C,hypothesis)\end{aligned} \tag{R81}$$

$$\begin{aligned}&\text{IF } detectionexp(D,C,\text{-}1)\\&\text{and } unspecificdetector(D,X)\\&\text{THEN } notlocalized(X,C,confirmed)\end{aligned} \tag{R82}$$

$$\begin{aligned}&\text{IF } onetomanyinteractionexp(X,S)\\&\text{THEN } complex(newcomplex(X,S))\\&\text{and } multiset(newmultiset(X,S))\\&\text{and } component(X,newcomplex(X,S),u)\\&\text{and } belong(X,newmultiset(X,S),u)\end{aligned} \tag{R83}$$

$$\begin{aligned}&\text{IF } onetomanyinteractionexp(X,S)\\&\text{and } belong(Y,S,N)\\&\text{THEN } component(Y,newcomplex(X,S),N)\\&\text{and } belong(Y,newmultiset(X,S),N)\end{aligned} \tag{R84}$$

$$\begin{aligned}&\text{IF } onetomanyinteractionexp(X,S)\\&\text{THEN } association(newmultiset(X,S),newcomplex(X,S),confirmed)\end{aligned} \tag{R85}$$

$$\begin{aligned}&\text{IF } onetooneinteractionexp(X,Y)\\&\text{THEN } complex(newcomplex(X,Y))\\&\text{and } multiset(newmultiset(X,Y))\\&\text{and } component(X,newcomplex(X,Y),u)\\&\text{and } component(Y,newcomplex(X,Y),u)\\&\text{and } belong(X,newmultiset(X,Y),u)\\&\text{and } belong(Y,newmultiset(X,Y),u)\\&\text{and } association(newmultiset(X,Y),newcomplex(X,Y))\end{aligned} \tag{R86}$$

$$\begin{aligned}&\text{IF } manyinteractionexp(S)\\&\text{THEN } complex(newcomplex(S))\\&\text{and } association(S,newcomplex(S),confirmed)\end{aligned} \tag{R87}$$

$$\begin{array}{l}\text{IF } manyinteractionexp(S)\\ \text{and } belong(X,S,N)\\ \text{THEN } component(X, newcomplex(S), N)\end{array} \tag{R88}$$

$$\begin{array}{l}\text{IF } effectononetomanyinteractionexp(Xa,Y,S,E)\\ \text{THEN } complex(newcomplex(Y,S))\\ \text{and } multiset(newmultiset(Y,S))\\ \text{and } component(Y, newcomplex(Y,S))\\ \text{and } belong(Y, newmultiset(Y,S))\end{array} \tag{R89}$$

$$\begin{array}{l}\text{IF } effectononetomanyinteractionexp(Xa,Y,S,E)\\ \text{and } belong(Z,S,N)\\ \text{THEN } component(Z, newcomplex(Y,S), N)\\ \text{and } belong(Z, newmultiset(Y,S), N)\end{array} \tag{R90}$$

$$\begin{array}{l}\text{IF } effectononetomanyinteractionexp(Xa,Y,S,E)\\ \text{THEN } association(newmultiset(Y,S), newcomplex(Y,S), confirmed)\\ \text{and } modulatesassociation(Xa, newmultiset(Y,S), newcomplex(Y,S), E, unknown, confirmed)\end{array} \tag{R91}$$

$$\begin{array}{l}\text{IF } sea(Xa,Ya,Sr,Sp,E)\\ \text{and } catalyzes(Ya,Sr,Sp,confirmed)\\ \text{and } modifiedform(Ya,Y)\\ \text{THEN } modulates(Xa,Y,Ya,E,unknown,confirmed)\end{array} \tag{R92}$$

Analytical rules

$$\begin{array}{l}\text{IF } phosphoform(Xa,X,Pos)\\ \text{THEN } modifiedform(Xa,X)\end{array} \tag{R93}$$

$$\begin{array}{l}\text{IF } acetylform(Xa,X,Pos)\\ \text{THEN } modifiedform(Xa,X)\end{array} \tag{R94}$$

$$\begin{array}{l}\text{IF } modifiedform(Xa,X)\\ \text{THEN } modifiedform(X,Xa)\end{array} \tag{R95}$$

$$\begin{array}{l}\text{IF } ko(K,X)\\ \text{and } modifiedform(X,Xa)\\ \text{THEN } ko(K,Xa)\end{array} \tag{R96}$$

$$\begin{array}{l}\text{IF } sirnaagainst(S,X)\\ \text{and } modifiedform(X,Xa)\\ \text{THEN } sirnaagainst(S,Xa)\end{array} \tag{R97}$$

$$\text{IF } \mathit{panantibodyagainst}(A,X) \text{ and } \mathit{modifiedform}(X,Xa) \text{ THEN } \mathit{panantibodyagainst}(A,Xa) \tag{R98}$$

$$\text{IF } \mathit{modulates}(X,Y,Ya,E,D,S) \text{ THEN } \mathit{modifiedform}(Ya,Y) \tag{R99}$$

$$\text{IF } \mathit{modulatesassociation}(X,Y,Ya,E,D,S) \text{ THEN } \mathit{modifiedform}(Ya,Y) \tag{R100}$$

$$\text{IF } \mathit{modulatesdissociation}(X,Y,Ya,E,D,S) \text{ THEN } \mathit{modifiedform}(Ya,Y) \tag{R101}$$

$$\text{IF } \mathit{modulates}(Xa,Y,Ya,E1,D1,\mathit{confirmed}) \text{ and } \mathit{modulates}(Ya,Z,Za,E2,D2,\mathit{confirmed}) \text{ THEN } \mathit{modulates}(Xa,Z,Za,E1*E2,\mathit{indirect},\mathit{confirmed}) \tag{R102}$$

$$\text{IF } \mathit{catalyzes}(Xa,Sr,Sp,\mathit{confirmed}) \text{ and } \mathit{belong}(Y,Sr,Ny) \text{ and } \mathit{belong}(Z,Sp,Nz) \text{ THEN } \mathit{modulates}(Xa,Y,Z,1,\mathit{direct},\mathit{confirmed}) \tag{R103}$$

$$\text{IF } \mathit{modulates}(Xa,Z,Za,1,\mathit{unknown},\mathit{confirmed}) \text{ and } \mathit{modulates}(Ya,Z,Za,1,\mathit{direct},\mathit{confirmed}) \text{ and } \mathit{modifiedform}(Ya,Y) \text{ THEN } \mathit{modulates}(Xa,Y,Ya,1,\mathit{unknown},\mathit{hypothesis}) \tag{R104}$$

$$\text{IF } \mathit{functionalequivalent}(Xa,X) \text{ and } \mathit{modulates}(Xa,Y,Ya,E,D,S) \text{ THEN } \mathit{modulates}(X,Y,Ya,E,D,S) \tag{R105}$$

$$\text{IF } \mathit{functionalequivalent}(Xa,X) \text{ and } \mathit{modulatestranslation}(Xa,Y,Ya,E,D,S) \text{ THEN } \mathit{modulatestranslation}(X,Y,Ya,E,D,S) \tag{R106}$$

$$\text{IF } \mathit{functionalequivalent}(Xa,X) \text{ and } \mathit{modulatestranscription}(Xa,Y,Ya,E,D,S) \text{ THEN } \mathit{modulatestranscription}(X,Y,Ya,E,D,S) \tag{R107}$$

$$\text{IF } \mathit{functionalequivalent}(Xa,X) \text{ and } \mathit{modulatesdissociation}(Xa,Y,Ya,E,D,S) \text{ THEN } \mathit{modulatesdissociation}(X,Y,Ya,E,D,S) \tag{R108}$$

$$\text{IF } \mathit{functionalequivalent}(Xa,X) \text{ and } \mathit{modulatesassociation}(Xa,Y,Ya,E,D,S) \text{ THEN } \mathit{modulatesassociation}(X,Y,Ya,E,D,S) \tag{R109}$$

Example of deduction

Let us consider the experimental results formalized by the following facts:

- $pa(fsh, erk, a\_perk, increase)$: in the presence of FSH the signal detected by the antibody *a_perk* increases;
- $pa(fsh, mek, a\_pmek, increase)$: in the presence of FSH the target molecule detected by the antibody *a_pmek* increases;
- $icppa(fsh, erk, a\_perk, pd98059, decrease)$: in the presence of FSH and *pd98059*, a specific inhibitor of MEK, the target molecule detected by the antibody *a_perk* decreases as compared to the condition with FSH alone;

and the following background facts:

- $modifiedForm(pmek, mek)$: phospho-MEK is a modified form of MEK;
- $modifiedForm(perk, erk)$: phospho-ERK is a modified from of ERK;
- $antibodyAgainst(a\_perk, perk)$: *a_perk* is an antibody targeting phospho-ERK;
- $antibodyAgainst(a\_pmek, pmek)$: *a_mek* is an antibody targeting phospho-MEK;
- $inhibitorAgainst(pd98059, pmek)$: PD98059 is an inhibitor of MEK.
- $notModified(fsh)$: FSH has no modified form.

We consider the following ontological rules:

$$\text{IF } inhibitorAgainst(I,X) \text{ THEN } specificDisruptor(I,X) \tag{R110}$$

$$\text{IF } antibodyAgainst(A,X) \text{ THEN } specificDetector(A,X) \tag{R111}$$

$$\text{IF } pa(X,Y,D,E) \text{ THEN } simplePa(X,Y,D,E) \tag{R112}$$

$$\text{IF } icppa(X^a,Y,A,I,E) \text{ THEN } complexPa(X^a,Y,A,I,E) \tag{R113}$$

and the following interpretative rules:

$$\begin{aligned} &\text{IF } simplePa(X,Y,D,E) \\ &\text{and } specificDetector(D,Y^a) \text{ and } modifiedForm(Y^a,Y) \\ &\text{THEN } modulates(X,Y,Y^a,E,unknown,confirmed) \end{aligned} \tag{R114}$$

$$\begin{aligned} &\text{IF } complexPa(X^a,Y,A,I,decrease) \\ &\text{and } specificDetector(A,Z^a) \text{ and } specificDisruptor(I,Y^a) \\ &\text{and } modifiedForm(Y^a,Y) \\ &\text{and } notModified(X^a) \\ &\text{and } modulates(X^a,Z,Z^a,increase,unknown,confirmed) \\ &\text{THEN } modulates(Y^a,Z,Z^a,increase,confirmed) \\ &\text{and } modulates(X^a,Y,Y^a,increase,unknown,hypothesis) \end{aligned} \tag{R115}$$

Three successive deduction steps allow deducing new modulations from the three experimental results.

First, using ontological rules for experimental result predicates (rules (R112), (R113)) and ontological rules for detectors and disruptors (rules (R110), (R111)), we deduce the following facts:

- $simplePa(fsh, mek, a\_pmek, increase)$
- $simplePa(fsh, erk, a\_perk, increase)$
- $complexPa(fsh, erk, a\_perk, pd98059, decrease)$
- $specificDetector(a\_perk, perk)$
- $specificDetector(a\_pmek, pmek)$
- $specificDisruptor(pd98059, pmek)$.

Second, we use the simple rule (R114) to interpret the two *pa* predicates, deducing two new modulations:

- $modulates(fsh, mek, pmek, increase, unknown, confirmed)$: FSH stimulates the phosphorylation of MEK;
- $modulates(fsh, erk, perk, increase, unknown, confirmed)$: FSH stimulates the phosphorylation of ERK.

Finally, using this last fact and the complex rule (R115), we can interpret the *icppa*, and deduce the following modulations:

- $modulates(pmek, erk, perk, increase, unknown, confirmed)$: phospho-MEK stimulates the phosphorylation of ERK
- $modulates(fsh, mek, pmek, increase, unknown, hypothesis)$: FSH may stimulate the phosphorylation of phospho-MEK (hypothesis).

Example of abduction

We make the hypothesis that phospho-MEK stimulates the phosphorylation of ERK, and we want to obtain experimental plans in order to test this hypothesis. The abductive observation built from this hypothesis is the following fact:

$$modulates(pmek, erk, perk, increase, unknown, confirmed) \quad \text{(F1)}$$

In this example, we consider the following ontological rules:

$$\text{IF } inhibitorAgainst(I, X) \text{ THEN } specificDisruptor(I, X) \quad \text{(R116)}$$

$$\text{IF } antibodyAgainst(A, X) \text{ THEN } specificDetector(A, X) \quad \text{(R117)}$$

$$\text{IF } pa(X, Y, D, E) \text{ THEN } simplePa(X, Y, D, E) \quad \text{(R118)}$$

$$\text{IF } icppa(X^a, Y, A, I, E) \text{ THEN } complexPa(X^a, Y, A, I, E) \quad \text{(R119)}$$

$$\text{IF } pra(X, Y, D, E) \text{ THEN } simplePa(X, Y, D, E) \quad \text{(R120)}$$

and the following interpretative rules:

$$\begin{array}{l} \text{IF } simplePa(X, Y, D, E) \\ \text{and } specificDetector(D, Y^a) \text{ and } modifiedForm(Y^a, Y) \\ \text{THEN } modulates(X, Y, Y^a, E, unknown, confirmed) \end{array} \quad \text{(R121)}$$

$$\begin{aligned}
&\text{IF } complexPa(X^a, Y, A, I, decrease)\\
&\quad\text{and } specificDetector(A, Z^a) \text{ and } specificDisruptor(I, Y^a)\\
&\quad\text{and } modifiedForm(Y^a, Y)\\
&\quad\text{and } notModified(X^a)\\
&\quad\text{and } modulates(X^a, Z, Z^a, increase, unknown, confirmed)\\
&\text{THEN } modulates(Y^a, Z, Z^a, increase, confirmed)\\
&\quad\text{and } modulates(X^a, Y, Y^a, increase, unknown, hypothesis)
\end{aligned} \tag{R122}$$

We also consider the following background facts:

- $modifiedForm(pmek, mek)$
- $modifiedForm(perk, erk)$
- $antibodyAgainst(a\_perk, perk)$
- $antibodyAgainst(a\_pmek, pmek)$
- $radioLabeledForm(r\_erk, erk)$
- $inhibitor(pd98059, pmek)$

Finally, we limit the set of abducibles to the predicates that formalize results from the following experiment types: *pa*, *pra* and *icppa*. Thus, the abductive task aims at finding explanations for fact (F1), under the form of experimental results of phosphorylation assays: we want to know which phosphorylation assays should be made to prove our hypothesis.

There are two main possible explanations for this fact:

(1) Fact (F1) can be deduced from rule (R121) if

$$simplePa(pmek, erk, a\_perk, increase) \tag{F2}$$

or

$$simplePa(pmek, erk, r\_perk, increase) \tag{F3}$$

hold. In turn, each of these two facts could be a consequence of rule (R118) or rule (R120), leading to four possible experimental results among which only two respect the typing constraints of the arguments of their associated predicates: the *pa* using the antibody and the *pra* using the radio labeled form. Hence, among simple assays, only the two following experimental results could explain fact (F1) alone:

$$pa(pmek, erk, a\_perk, increase) \tag{F4}$$

and

$$pra(pmek, erk, r\_perk, increase) \tag{F5}$$

(2) Fact (F1) could be a consequence of rule (R122), as long as there exists some molecule X such that

$$modulates(X, erk, perk, increase, unknown, confirmed) \tag{F6}$$

and

$$notModified(X) \tag{F7}$$

hold and at least one of the two following facts holds:

$$complexPa(X, erk, a\_perk, pd98059, decrease) \tag{F8}$$

and

$$complexPa(X, erk, r\_perk, pd98059, decrease) \tag{F9}$$

By reasoning as previously, we can conclude that the modulation formalized by fact (F6) could be explained by either of the two following simple assay results:

$$pa(X, erk, a\_perk, increase) \tag{F10}$$

and

$$pra(X, erk, r\_perk, increase) \tag{F11}$$

Two possible explanations remain, one for each of the two facts (F8) and (F9). Each of these two facts could be a consequence of the rule (R119) for *icppa*. Hence fact (F8) could be explained by

$$icppa(X, erk, a\_perk, pd98059, decrease) \tag{F12}$$

and fact (F9) by

$$icppa(X, mek, r\_perk, pd98059, decrease) \tag{F13}$$

This last experimental fact does not respect the typing constraints associated to the arguments of the *icppa* predicate, as it uses a radio-labeled form instead of an antibody as a detector. Hence it is not a suitable explanation to fact (F9). To summarize, our goal fact (F1) could be explained by fact (F6) and either of the two facts (F8) and (F9): there exists a molecule X that activates the phosphorylation of ERK, and in the presence of inhibitor PD98059, the phosphorylation induced by X of either ERK or MEK decreases. Fact (F6) can in turn be explained by either of the two simple assay results (F10) and (F11): the existence of X can be demonstrated by a phosphorylation assay, using an antibody or a radioactivity for detection. As for facts (F8) and (F9), the first one can be explained by the complex assay result (F12): a phosphorylation assay in the presence of the inhibitor PD98059 where the quantity of phospho-ERK is detected using a specific antibody; while the second one cannot be explained.

As a consequence of the inferences made in points (1) and (2), the following sets of experimental results, each corresponding to a different experiment plan, could explain our goal fact (F1):

- $\{pa(pmek, erk, a\_perk, increase)\}$;
- $\{pra(pmek, erk, r\_perk, increase)\}$;
- $\{pa(X, erk, a\_perk, increase), icppa(X, erk, a\_perk, pd98059, decrease)\}$, for some molecule X such that $notModified(X)$ holds;
- $\{pra(X, erk, r\_perk, increase), icppa(X, erk, a\_perk, pd98059, decrease)\}$, for some molecule X such that $notModified(X)$ holds.

The first two plans consist in checking whether the phosphorylation of ERK is stronger in the presence of phospho-MEK. Given that both are intracellular proteins, this would require making the experiment on purified proteins. The last two experimental plans consist in checking whether in the presence of some signal X, the quantity of phospho-ERK increases, and whether this effect is lower when adding the inhibitor PD98059.

Note that had we considered that we know that FSH has no modified form by adding the background fact $notModified(fsh)$ to the background theory, variable X would have been replaced by the constant *fsh* in the two last experimental plans, as follows:

- $\{pa(fsh, erk, a\_perk, increase), icppa(fsh, erk, a\_perk, pd98059, decrease)\}$;
- $\{pra(fsh, erk, r\_perk, increase), icppa(fsh, erk, a\_perk, pd98059, decrease)\}$.

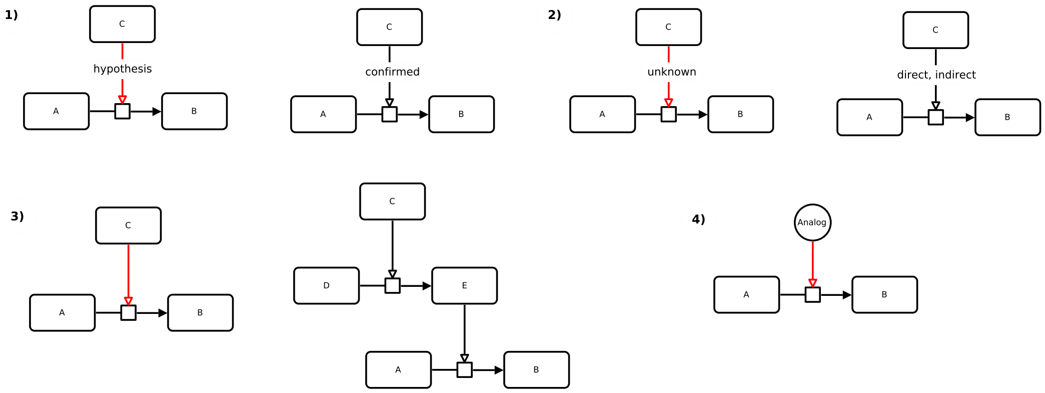

Supplementary Figure S1. Different cases of fact pruning for the G protein pathway. A deduced fact (in red) is pruned if and only if it belongs to one of the four following cases: 1) it represents a modulation with the status *hypothesis* and there exists the exact same fact in the set but with the status *confirmed*; 2) it represents a modulation with the distance *unknown* and there exists the exact same fact in the set but with the distance *direct* or *indirect*; 3) it represents a modulation that can be deduced by transitivity from other facts of the set representing modulations; 4) it represents a modulation by a functional analog that does not belong to the network.

Comparison of the G protein pathway

Amongst our deduced facts, 113 were related to the G protein pathway. From this initial subset of facts, we built a new set, denoted by F_{DED}, by removing facts that were irrelevant for the comparison, in two phases. First, we removed all references to phosphorylation sites from the facts of the initial subset, as the network from the literature did not mention any sites of phosphorylation. Then, we pruned the obtained set by removing all facts that were not considered as meaningful, i.e. for which there exist another fact in the set that is more precise; or that can be deduced by transitivity; or that bring into play a functional analog or a perturbator that should not appear in the network; or that formalize the fact that some molecule has no effect on a process. More precisely, a fact of the initial set was labeled as not meaningful if and only if:

1. it represents a modulation with the status *hypothesis* and there exists the exact same fact in the set but with the status *confirmed*;

2. it represents a modulation with the distance *unknown* and there exists the exact same fact in the set but with the distance *direct* or *indirect*;

3. it represents a modulation that can be deduced by transitivity from other facts of the set representing modulations;

4. it represents a modulation by a signal or a perturbator (for example an inhibitor or a functional analog) which should not appear in the final network, since it was added by the experimenter but is normally not present in the cell;

5. it represents a modulation with the effect *noeffect*.

These different cases (but the last one) are shown in figure 1.

These two transformation steps allowed obtaining a set of 73 facts, 54 of them having the status *confirmed*, and the rest having the status *hypothesis*. We compared this set of facts to the set of 20 facts, denoted by F_{LIT}, that we obtained by translating the literature network of the G protein pathway[1]. Seven facts belonged to both F_{DED} and F_{LIT}. These facts represent the modulation of the phosphorylation of ERK1 or ERK2 by phospho-MEK1 or phospho-MEK2, the catalysis of ATP into cyclic AMP (cAMP) by adenylyl cyclase (AC), the catalysis of cAMP into AMP by a phosphodiesterase (PDE), and the modulation of the phosphorylation of p38MAPK by protein kinase A (PKA).

One could be surprised by the fact that the majority of facts in F_{DED} are not present in F_{LIT}. We distinguish the following cases to explain the presence of facts in F_{DED} that are not in F_{LIT}:

- Some facts of F_{DED} are not present in F_{LIT} but are implicit in the literature network. For example, a fact of F_{LIT} formalizes the complexation of GTP with protein Gαs. The complexation is not represented in the literature network, and thus is not formalized by any fact in F_{LIT}. However, the complex GTP-Gαs is present in the literature network, and the complexation process is thus implicitly present in this network.

- Some facts of F_{DED} can be deduced by transitivity from facts of F_{LIT}. For example, a fact of F_{DED} formalizes the stimulation of the phosphorylation of ERK1,2 by the complex GTP-Gαs. This stimulation is not represented in the literature network, but it could be deduced by transitivity from the effect of GTP-Gαs on the cAMP/PKA/Raf1/MEK1,2/ERK1,2 pathway.
- Some facts of F_{DED} express relations that are more precise than those represented in the literature network. For example, a fact of F_{DED} formalizes the stimulation of the complexation between Rap1 and GTP by the complex cAMP-EPAC. This stimulation is not represented as such in the literature network, where it is the active form of EPAC that stimulates this complexation. However, we know that the active form of EPAC is indeed the complex cAMP-EPAC. Thus, in the literature network, the mechanism of activation of EPAC is not shown, whereas it is formalized by a fact of F_{DED}.
- Some facts of F_{DED} have the *hypothesis* status, whereas they have the *confirmed* status in F_{LIT}. It is for example the case of the fact expressing the stimulation of the process activating PKA, by cAMP. This is a well known activation process, however, it has not been shown in the precise case of the FSHR, and thus was not present in our experimental facts corpus.
- Some facts of F_{DED} have been deduced using experimental results that have not been taken into account for the construction of the literature network, being judged as untrustworthy. These facts were taken into account for the automatic construction in order to deduce as many facts as possible. This is for example the case of the stimulation of the phosphorylation of p38MAPK by MEK2.

As for the 13 facts that are in F_{LIT} but not in F_{DED}, we observe the two followings cases:

- Some facts of F_{LIT} formalize relations that are more precise than facts of F_{DED}. For example, a fact of F_{LIT} formalizes the stimulation of the process activating PDE, by the complex FSH-FSHR-Gαs-GTP. Yet, in F_{DED}, a fact formalizes the same stimulation but by the complex FSH-FSHR alone. The activation of Gαs by FSHR is well known, but the numerous publications demonstrating it were not included in the corpus.
- Some facts of F_{LIT} have not been proved for the FSHR induced network but for other GPCRs. Indeed, it is largely admitted that some knowledge can be transferred from a receptor to another.

Traces in ASP

An ASP rule is of the following form:

$$H\text{:-}B_1;\ldots;B_k;not\ B_{k+1};\ldots;not\ B_n.$$

where H and all B_is are atoms (i.e. a predicates applied to a tuple of terms). H is called the head of the rule, while the set of B_is is called its body. A rule with an empty body is a fact, while a rule with an empty head is a constraint. An ASP rule should be read as: if the body of a rule holds, then the head of that rule should hold.

A set of ASP rules forms a logic program. Given a logic program modeling a problem, all solutions (of the problem) can be found by computing the answer sets of that program, that are usually the stables models of that program.

Deduction is realized as follows: Given a background theory formed of our deductive rules and a set of facts representing background knowledge and experimental results, we build a logic program formed of ASP rules. Deductive rules and facts can be straightforwardly encoded into ASP rules. Since the obtained logic program has no negation, it has only one answer set, that is exactly the set of facts that can be deduced from the background theory. Hence, we obtain all deduced facts by computing the unique answer set of the program, using software clingo.

Traces of facts can be obtained by associating a name to each rule and by rewriting each rule into two new rules. We illustrate this rewriting on an example.

Let $a(X,Z)\text{:-}b(X,Y);c(Y,Z)$. be a rule named $r1$. Rule $r1$ is rewritten into the two following rules: $r1(X,Y,Z)\text{:-}b(X,Y);c(Y,Z)$. and $a(X,Z)\text{:-}r1(X,Y,Z)$.. Intuitively, in the first rule, predicate $r1(X,Y,Z)$ allows to keep trace of the use of rule $r1$: $r1(X,Y,Z)$ is deduced if and only if rule $r1$ could have been triggered in the program without rewriting, and predicate $r1$ contains all arguments that occur in the body of rule $r1$. The second rule allows to deduce predicate $a(X,Z)$ exactly as with $r1$. Rewriting all rules with intermediaries in the form of predicates r_is allows to keep trace of the rules that are used to deduce facts in a simple manner.

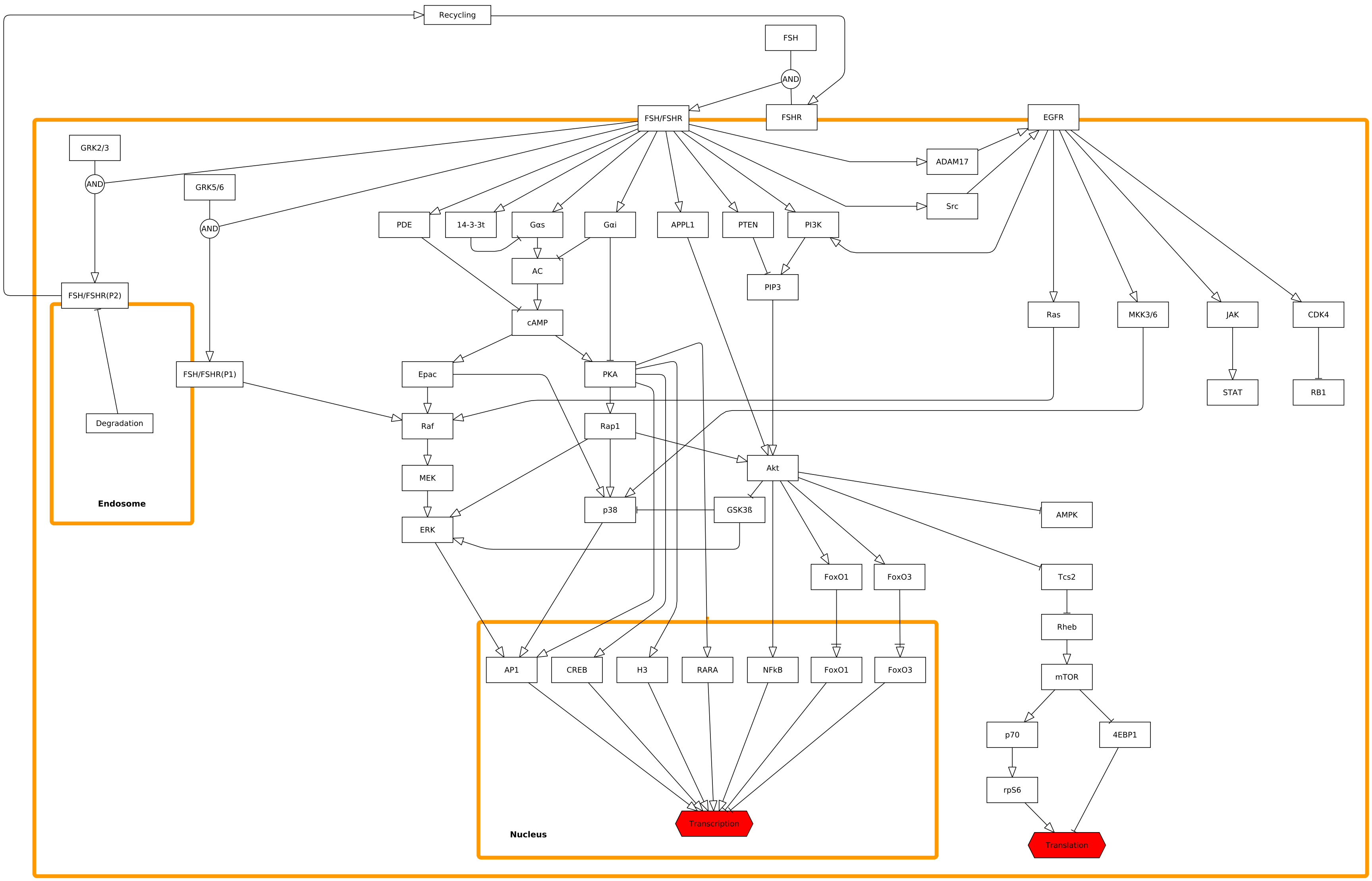

Supplementary Figure S2. SBGN AF map of the different signaling pathways triggerd by the FSHR.

References

1. Gloaguen, P., Crépieux, P., Heitzler, D., Poupon, A. & Reiter, E. Mapping the follicle-stimulating hormone-induced signaling networks. *Front. endocrinology* **2**, 45 (2011).
